# Construction of gateway-compatible baculovirus expression vectors for high-throughput protein expression and in vivo microcrystal screening

**DOI:** 10.1038/s41598-020-70163-2

**Published:** 2020-08-07

**Authors:** Yanyang Tang, Justin Saul, Nirupa Nagaratnam, Jose M. Martin-Garcia, Petra Fromme, Ji Qiu, Joshua LaBaer

**Affiliations:** 1 School of Molecular Sciences, Arizona State University, Tempe, AZ 85287 USA; 2 Virginia G. Piper Center for Personalized Diagnostics, The Biodesign Institute, Arizona State University, Tempe, AZ 85281 USA; 3 Center for Applied Structural Discovery, The Biodesign Institute, Arizona State University, Tempe, AZ 85281 USA

**Keywords:** X-ray crystallography, Expression systems, High-throughput screening

## Abstract

Baculovirus mediated-insect cell expression systems have been widely used for producing heterogeneous proteins. However, to date, there is still the lack of an easy-to-manipulate system that enables the high-throughput protein characterization in insect cells by taking advantage of large existing Gateway clone libraries. To resolve this limitation, we have constructed a suite of Gateway-compatible pIEx-derived baculovirus expression vectors that allow the rapid and cost-effective construction of expression clones for mass parallel protein expression in insect cells. This vector collection also supports the attachment of a variety of fusion tags to target proteins to meet the needs for different research applications. We first demonstrated the utility of these vectors for protein expression and purification using a set of 40 target proteins of various sizes, cellular localizations and host organisms. We then established a scalable pipeline coupled with the SONICC and TEM techniques to screen for microcrystal formation within living insect cells. Using this pipeline, we successfully identified microcrystals for ~ 16% of the tested protein set, which can be potentially used for structure elucidation by X-ray crystallography. In summary, we have established a versatile pipeline enabling parallel gene cloning, protein expression and purification, and in vivo microcrystal screening for structural studies.

## Introduction

Proteins orchestrate the biological processes in living organisms through their interactions or manipulations of other biomolecules. Central to understanding the molecular function of a protein is to elucidate its three-dimensional (3D) structure, which requires expression and purification of large quantities of highly pure and properly folded protein^1,2^. Yet, in the current proteomic era, the rapidly increasing demand for stable and functional proteins for research and commercial uses is still orders of magnitude higher than the available supply^3,4^, which largely hinders protein characterization, structure determination as well as the development of protein-based therapeutics.

The conventional approach to determining protein structures by X-ray crystallography relies heavily on the complicated and tedious screening of appropriate conditions for the growth of sufficiently large and well-ordered protein crystals, which emphasizes one of the major bottlenecks of structural biology. Heterogeneously expressed proteins in the baculovirus-insect cell system can spontaneously form crystals within living cells, although this was commonly perceived as a somewhat rare event^5,6^. To date, a total of five recombinant proteins were reported to form the non-native microcrystals in baculovirus-infected insect cells, including (1) an artificial variant of the heterodimeric phosphatase calcineurin^7^, (2) firefly luciferase^8^, (3) inosine monophosphate dehydrogenase from *Trypanosoma brucei* (TbIMPDH)^9,10^, (4) glycosylated cysteine protease cathepsin B from *Trypanosoma brucei* (TbCatB)^11^, and (5) the avian reovirus μNS protein fused to GFP (GFP-μNS)^8^. Those crystals differ in many features including crystal morphology, stability, dimensions, growth dynamics, and subcellular localization^5^. The use of in vivo crystallography could eliminate the need for the extremely labor-intensive and time-consuming procedures associated with protein purification and in vitro crystallization. However, the number of protein structures available from in vivo-grown crystals has always been limited by their small size and their susceptibility to radiation damage^9,11,12^. These limitations have been recently overcome by the emergent technique of serial femtosecond crystallography (SFX) developed at X-ray free electron lasers (XFELs) as well as synchrotrons^10–13^, allowing data to be collected in a serial fashion from a stream of small nano- or micro-crystals for high-resolution structure determination^5,9–11^. This emerging concept of using serial crystallography with in vivo crystals opens new routes in structural biology of solving 3D protein structures^9,11^, and also highlights the significance of identifying novel in vivo crystal targets^12–14^. Thus, a high-throughput (HT) protein production pipeline built on the baculovirus-insect cell system will be extremely beneficial to the rapid screening for in vivo microcrystals that could be potentially advanced to serial crystallography for structure determination studies.

The baculovirus-mediated insect cell system has many advantages for protein expression—easy manipulation, low cost, accommodation of large DNA inserts, relatively high production level, and essential eukaryotic protein modifications similar to mammalian cells^15–17^. However, the procedures for inserting foreign genes into the baculoviral genome and repeated rounds of plaque purification necessary to isolate recombinants from the wild-type parental virus have been traditionally tedious, labor-intensive, and time-consuming^18,19^, which largely restricts its development for HT protein production^17^. A series of molecular cloning technologies have been introduced to the system to improve the recombination efficiency by modifying the baculoviral genomic DNA^16,20–22^, which eventually gave birth to several commercialized baculovirus expression vector systems (BEVS), such as BacPAK6 (TaKaRa), Bac-to-Bac (Invitrogen), flashBac (Oxford ET), and BacMagic (Novagen).

The BacPAK6 system developed a triple-digested baculoviral genome that can force the homologous recombination with a transfer plasmid (pBacPAK6 from TaKaRa) to knock in the target gene and simultaneously restore the *ORF162*, an essential viral gene that is partially deleted from this construct, so that only the recombinant viral genome can replicate in insect cells^23^. Although this strategy efficiently increases the percentage of the recombinants^17,24^, it still requires plaque purification and verification of the recombinant phenotypes following recombination.

In parallel with BacPAK6, the Bac-to-Bac system introduced the bacterial replicon and transposon attachment site to the baculoviral genome. This bacmid technology permits the propagation of the baculoviral genome in *E. coli* (DH10Bac) as well as the T7-mediated transposition of a target gene from the transfer plasmid (pFastBac from Invitrogen) to generate the recombinant bacmid^25^. Although this approach can produce recombinant virus with almost 100% efficiency^17^, it requires the time-consuming process of antibiotic selection and blue-white screening for the recombinant bacmid^24^, thus compromising its application in HT protein production and its amenability to automation. Additionally, a potential disadvantage of this system is the loss of target protein expression after serial passage of recombinant virus in insect cells^26^, and this might be associated with the genetic instability due to the presence of bacterial sequence retained in viral genome^27^.

These limitations were resolved by the more recently developed flashBac system^28^, which represents a combination of bacmid technology and in vivo recombination with a transfer plasmid (pOET from Oxford ET). Upon homologous recombination, the target gene replaces the bacterial sequence that may cause poor genetic stability, simultaneously restoring *ORF1629* essential for replication. As no further separation techniques are required, the time and complexity of producing recombinant virus are remarkably reduced, thus making it suitable for automated HT protein expression^17,28^. The BacMagic system follows the same cloning principle and its latest bacmid has been further modified with deletions of several non-essential genes^29,30^, such as chitinase (*chiA*), cathepsin (*v-cath*), *p10*, *p74*, and *p26*, which greatly improves the recombinant protein yield by reducing the protein degradation and increasing the recombinant biomass^24^. Given the above-mentioned advantages, the BacMagic-3 system along with the transfer plasmid pIEx (Novagen) were selected to build the protein production pipeline in this study. However, the insertion of target gene into the pIEx vector requires restriction and ligation cloning that is not compatible with HT screening of many expression constructs. To reduce the time and resources needed to generate recombinant baculovirus at large scale, Radner et al*.* developed a ligation independent cloning (LIC) variant of the pIEx vector that permits parallel LIC cloning and screening of expression constructs in insect cells^31^. However, either multiple rounds of subcloning or substantial preparation of inserts from a genomic or cDNA template are required to obtain appropriately prepared PCR products prior to their insertion into the pIEx vector^32^.

**Figure 1:**
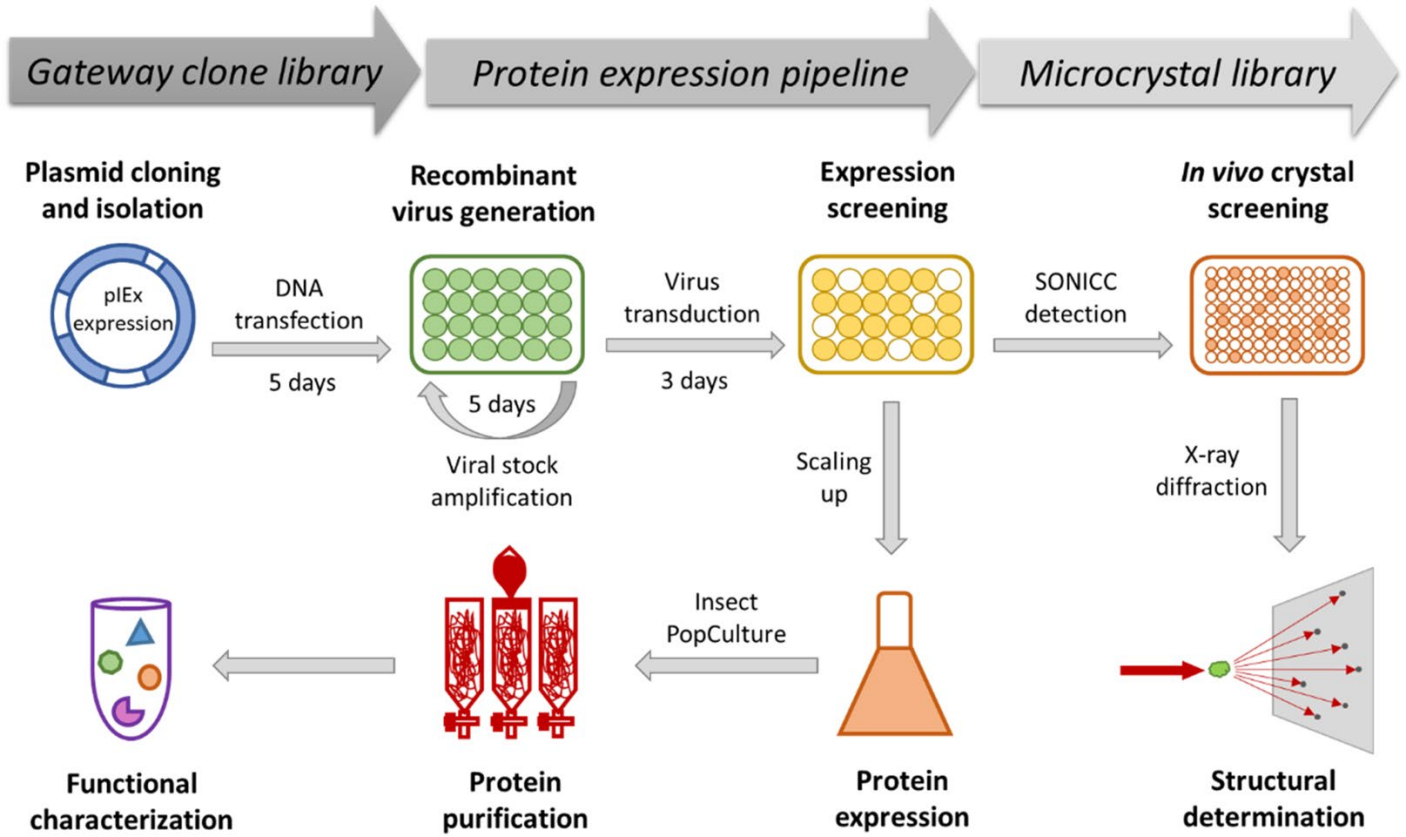
Workflow of the HT protein production and characterization pipeline. It enables: (1) easy selection of ORFs encoding target proteins from any existing Gateway clone libraries; (2) rapid and convenient HT construction of expression clones; (3) mass parallel expression screening of recombinant proteins with various tags; (4) affinity tag-based protein purification for functional characterization; and (5) fast and sensitive screening for in vivo microcrystal targets.

In the current study, we have developed a suite of Gateway-compatible variants of the pIEx vector such that any gene of interest in a Gateway donor clone can be transferred to these pIEx variants through one-step Gateway LR cloning to construct expression clones. Furthermore, these vectors were modified to contain the coding sequence of various fusion tags including EGFP, GFP, GST, His, FLAG, and HaloTag, at either the amino- (N-) or carboxy- (C-) terminus, to support recombinant protein expression with functional tags. In conjunction with our existing DNASU plasmid repository (https://dnasu.org/DNASU/Home.do), where thousands of open reading frames (ORFs) from many species (human, yeast, *Drosophila*, *Arabidopsis*, *Xenopus*, and many bacteria and viruses) are readily available in a Gateway donor clone^33^, we have established a reliable and versatile pipeline that enables mass parallel production of recombinant proteins fused to various tags for affinity purification and functional characterization (Fig. 1). Also, we have incorporated the Second Order Nonlinear Imaging of Chiral Crystals (SONICC) technology into our pipeline, which allows for highly sensitive screening for microcrystals (< 1 μm) in living cells in a HT fashion^34,35^. Together with confirmation by transmission electron microscopy (TEM), we have demonstrated the successful in vivo crystallization of 9 targets out of the 56 (~ 16%) target proteins tested.

## Results

### pIEx expression vector construction

**Figure 2:**
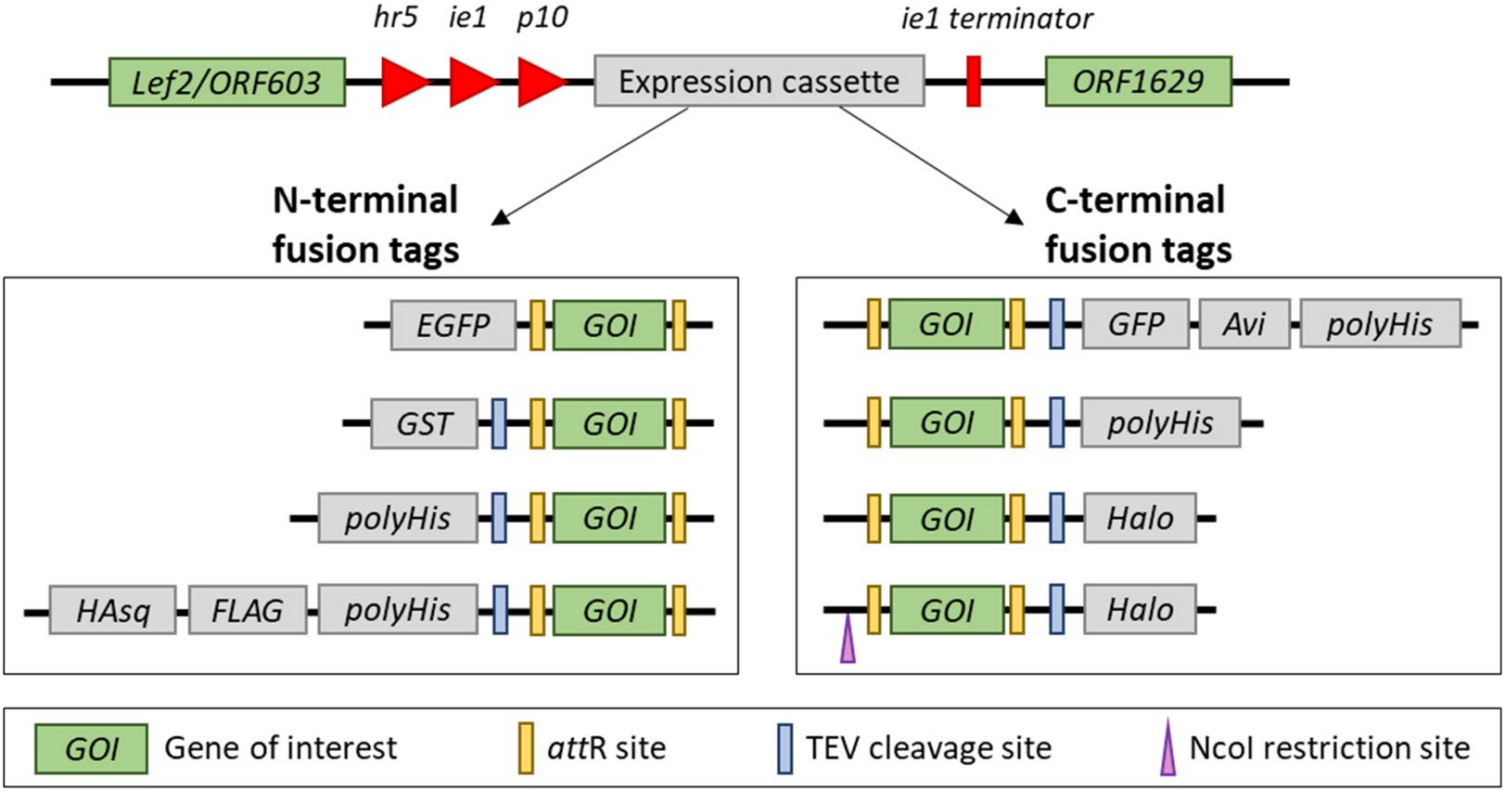
Schematic drawing of pIEx expression vectors. The pIEx expression vectors were derived from the pIEX-cyto vector containing baculovirus homologous region *Lef2/ORF603* and *ORF1629*, the enhancer *hr5* and early immediate promoter *ie1*. Through In-Fusion cloning, the Gateway death cassette flanked by *att*R recombination sites was introduced downstream of the baculovirus very late promoter *p10* and upstream of the *ie1* terminator. Sequences encoding fusion tags were inserted in the appropriate reading frame as indicated either upstream or downstream of the death cassette, which is later replaced by the gene of interest (GOI) upon Gateway LR reaction with the donor clone. An NcoI restriction site that contains ATG start codon initiates translation before the *att*R1 site.

**Figure 3:**
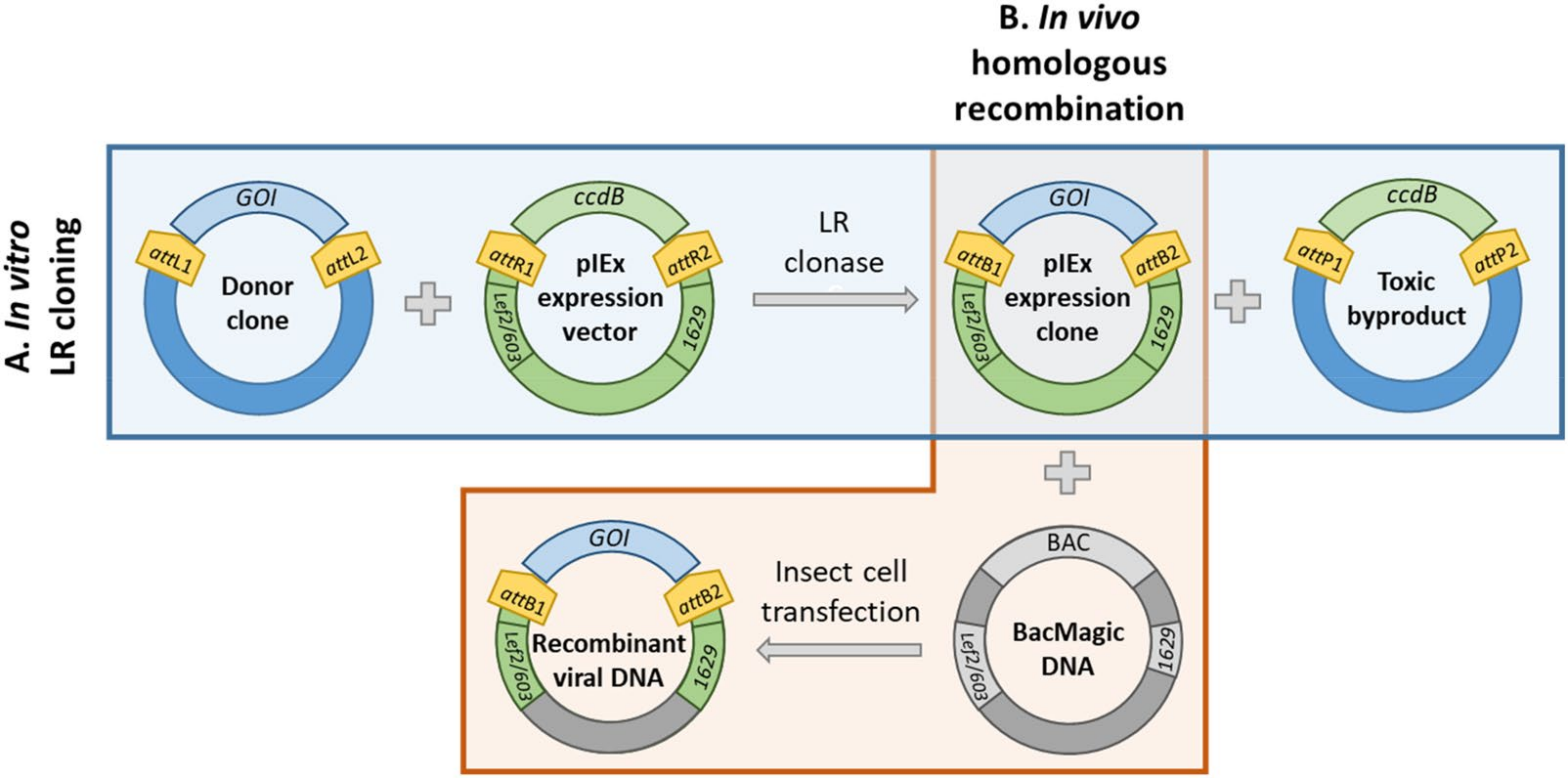
Working principle of the Gateway-compatible pIEx/BacMagic-3 expression system. (**A**) A gene of interest (GOI) in a donor clone is transferred to replace the death cassette containing the *ccdB* lethal gene in pIEx expression vector via Gateway recombination between *att*L and *att*R sites, producing a pIEx expression clone. (**B**) Sf9 cells are co-transfected with a pIEx expression clone and BacMagic-3 DNA, where the expression cassette flanked by *Lef2/ORF603* and *ORF1629* is integrated into recombinant viral DNA through homologous recombination, thus replacing the bacteria artificial chromosome (BAC) and repairing the defective gene *ORF1629*.

A suite of pIEx-based expression vectors for baculovirus-insect cell system have been generated to enable the rapid cloning of target genes into expression clones using Gateway technology. To construct a Gateway-compatible pIEx expression vector, the Gateway death cassette containing the *ccdB* lethal gene flanked by bacteriophage l site-specific recombination sites (*att*R1 and *att*R2) was introduced downstream of the very late *p10* promoter in the pIEx-cyto backbone (Fig. 2). When mediated by LR clonase, target genes in frame from a Gateway donor clone replace the death cassette in pIEx expression vectors, resulting in pIEx expression clones (Fig. 3A). Upon co-transfecting Sf9 cells with a pIEx expression clone and BacMagic-3 viral DNA, homologous recombination at viral-specific sequences (*Lef2/ORF603* and *ORF1629*) occurs, which consequently inserts the expression cassette (i.e. the target gene and fusion tag) and restores the essential gene *ORF1629* flanking the insertion site to eventually form recombinant viral DNA (Fig. 3B).

In addition, a series of fusion tags were introduced into the pIEx vector collection to support the expression of fusion proteins with nEGFP, cGFP-Avi-His, cHalo, nHis, cHis, nGST, or nHA secretory sequence (sq)-FLAG-His tags (Fig. 2, Table 1). Thus, this pIEx vector collection provides a variety of options to tag target proteins depending on the desired downstream applications. Particularly, there are two variants of pIEx expression vector with the cHalo tag: one initiates translation at the start codon before the *att*R1 site (named “pIEx-NcoI-cHalo”) and the other supports the translation initiation using the natural start codon located within the ORF insert following the *att*R1 site (named “pIEx-cHalo”) (Fig. 2). Additionally, a tobacco etch virus (TEV) protease cleavage site was also introduced before or after the C-terminal or N-terminal tag so that, if desired, the fusion tags can be readily removed from the recombinant proteins with TEV protease (Fig. 2).

**Table 1:** Gateway-compatible pIEx expression vector collection.

Vector	Gateway cloning	Fluorescent imaging	Purification	TEV cleavage	Translation start site
pIEx-nEGFP	✓	✓			Fusion tag
pIEx-cHalo	✓	✓^a^	✓	✓	Inserted ORF
pIEx-nGST	✓		✓	✓	Fusion tag
pIEx-nHis	✓		✓	✓	Fusion tag
pIEx-nHAsq-FLAG-His (pIEx-nHFH)	✓	✓^b^	✓	✓	Fusion tag
pIEx-cGFP-Avi-His (pIEx-cGAH)	✓	✓	✓	✓	Inserted ORF
pIEx-cHis	✓		✓	✓	Inserted ORF
pIEx-NcoI-cHalo (pIEx-N-cHalo)	✓	✓^a^	✓	✓	Gateway junction

^a^Via HaloTag fluorescent ligand (Promega) staining.

^b^Via immunofluorescence staining.

### High-throughput protein expression analysis

**Figure 4:**
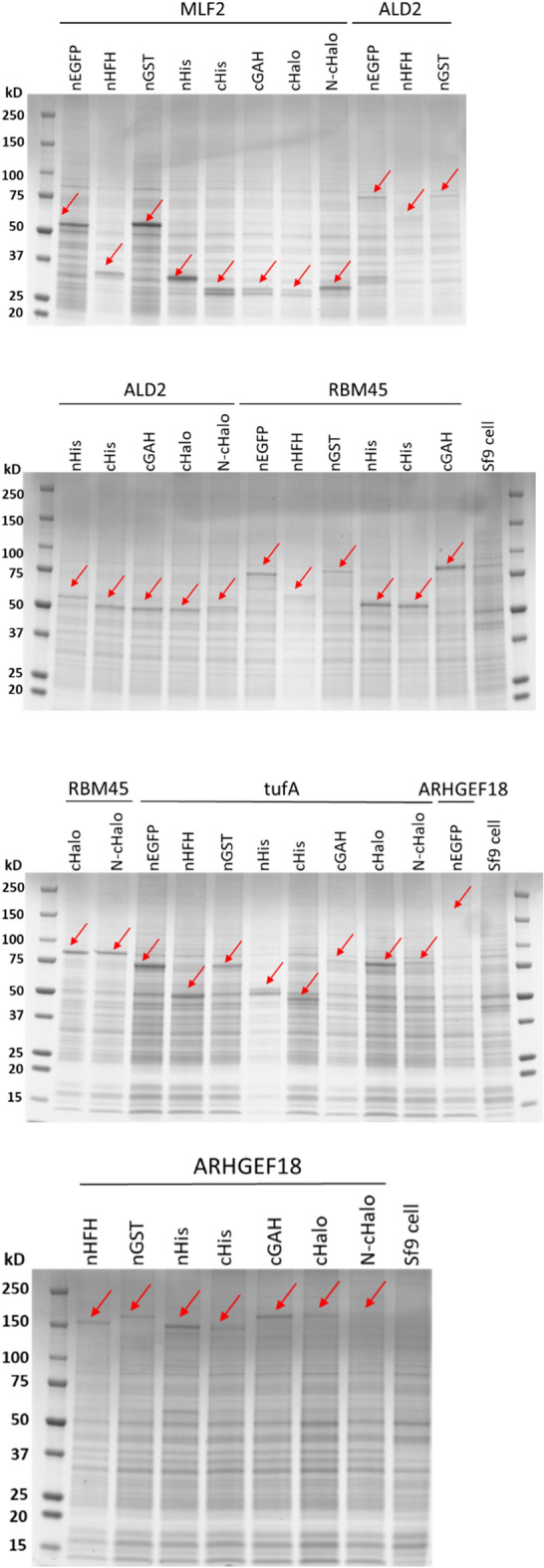
Successful parallel expression of target proteins. Target proteins were expressed using the indicated pIEx expression vectors and analyzed by Coomassie-stained SDS-PAGE. A target was considered as “expressed” if a novel band (red arrows) was present at the expected molecular weight but absent in the non-infected Sf9 cells. As expected, the two closed format clones, MLF2 and ALD2, were attached with no fusion tags when expressed using pIEx vectors carrying C-terminal tags.

To demonstrate the mass parallel expression of recombinant proteins, a test collection of ORFs encoding 40 individual full-length proteins in Gateway donor clones were transferred into the pIEx expression vectors (Supplementary Table S1). These target proteins were selected from a diverse range of molecular weight (8–130 kDa) and host organisms, including human, fungus, bacteria, and virus (Supplementary Fig. S1). Additionally, these proteins are located in different subcellular compartments of their original organisms as well as involved in various biological processes. Successful parallel protein expression from each pIEx expression vector was demonstrated by SDS-PAGE (Supplementary Fig. S2). For proof-of-principle, selected proteins were tested in certain vectors (Supplementary Table S2). Notably, five target proteins, including ALD2, MLF2, ARHGEF18, tufA, and RBM45, were successfully expressed in all pIEx expression vectors (Fig. 4). These 5 target proteins, including 3 from human, 1 from *Saccharomyces cerevisiae*, and 1 from *Vibrio cholerae*, range in size from ~ 25 to 130 kDa (Supplementary Table S2). These results indicate that the protein production pipeline built on the suite of Gateway-compatible pIEx expression vectors can achieve rapid mass parallel cloning and expression of recombinant proteins with various fusion tags.

To assess the reproducibility of protein production using our pipeline, expression assays were performed repeatedly for pIEx expression clones encoding nEGFP-WWTR1, nEGFP-MLF2, nEGFP-RGS13, and nEGFP-STAT4. SDS-PAGE analysis of Sf9 cells infected with recombinant viruses from different batches showed similar expression levels (Supplementary Fig. S3), suggesting that the pipeline is capable of producing recombinant proteins stably and reproducibly.

### Protein purification with fusion tags

**Figure 5:**
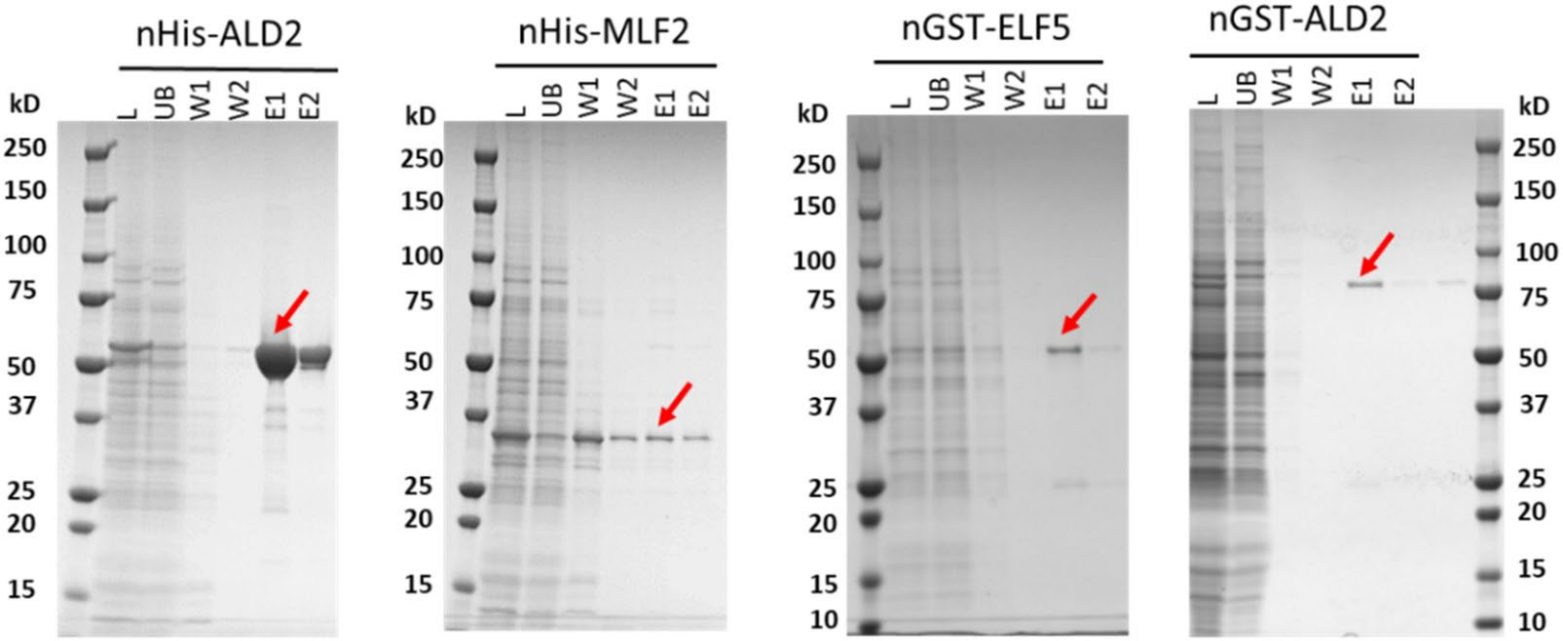
Protein purification. Recombinant proteins fused to nHis or nGST tags were successfully purified (red arrows) and analyzed by Coomassie-stained SDS-PAGE. *L* lysate, *UB* unbound, *W* wash, *E* elution.

As examples to demonstrate the successful purification of recombinant proteins overexpressed in Sf9 cells using our new pIEx vectors, several proteins were isolated from the Sf9 cell cultures expressing His-tagged or GST-tagged proteins. Specifically, His-tagged MLF2 and ALD2 recombinant proteins were successfully purified from expression constructs, pIEx-nHis-MLF2 and pIEx-nHis-ALD2, respectively, using nickel-nitrilotriacetic acid (Ni-NTA) agarose. Similarly, GST-tagged ALD2 and ELF5 recombinant proteins were successfully purified from expression constructs, pIEx-nGST-ALD2 and pIEx-nGST-ELF5, respectively, using magnetic glutathione resin (Fig. 5).

As protein solubility is crucial to purification and crystallization, a subset of targets was selected to characterize the solubility of recombinant proteins, including 8 targets for pIEx-nEGFP, 6 for pIEx-cHalo, 6 for pIEx-cGAH, 6 for pIEx-nHis, 5 for pIEx-cHis, 6 for pIEx-nGST, 6 for pIEx-nHFH, and 5 for the pIEx-NcoI-cHalo vector (Supplementary Table S3). Small-scale protein purification of cell extracts was performed for each of the pIEx expression vectors. The purified fraction was analyzed by Western blot using appropriate anti-fusion tag antibodies to confirm the identity of these targets. Out of 48 tested proteins, 24 were successfully purified and detected (Supplementary Fig. S4), suggesting that 50% of target proteins in the tested subset were soluble.

### SONICC screening for in vivo-grown microcrystals

To explore the feasibility of employing our tools in a HT structural biology pipeline using in vivo crystals, SONICC screening was performed to test for in vivo crystallization of recombinant proteins expressed using our pipeline. The SONICC method detects the presence of crystals as small as 100 nm of chiral molecules by second harmonic generation (SHG)^34^. When two infrared (IR) photons at 1,024 nm hit a chiral crystal with < 10 fs time difference, frequency doubling occurs by SHG whereby the crystal emits a green photon. Protein crystals in living insect cells are thereby detected by the green photons emitted. For amorphous precipitates or proteins in solution, the second harmonic signals cancel out. The SONICC measurements can be carried out in 96-well plates and thereby can be performed in a HT fashion.

As nEGFP-µNS has been reported to form in vivo crystals in living insect cells^8^, we included this target in our study as a positive control. In agreement with Schönherr et al*.*, we observed a strong intrinsic tendency of crystallization for nEGFP-µNS expressed in Sf9 cells (Supplementary Fig. S5). Around 72 h post infection (p.i.), the accumulation of rod-shaped structures, which were developed from the tiny spots representing the initial crystal nuclei, became visible within Sf9 cells under ultraviolet (UV) fluorescence microscopy (Supplementary Fig. S5A). In contrast, no fluorescent particles nor crystals were detected in cells expressing only the EGFP tag (Supplementary Fig. S5A). In addition, positive SONICC signal was detected in cells expressing nEGFP-μNS but not in non-infected cells (Supplementary Fig. S5B). Further investigation on the formation of nEGFP-µNS microcrystals and their diffraction characterization can be found in the recent report by Nagaratnam et al.^36^.

**Figure 6:**
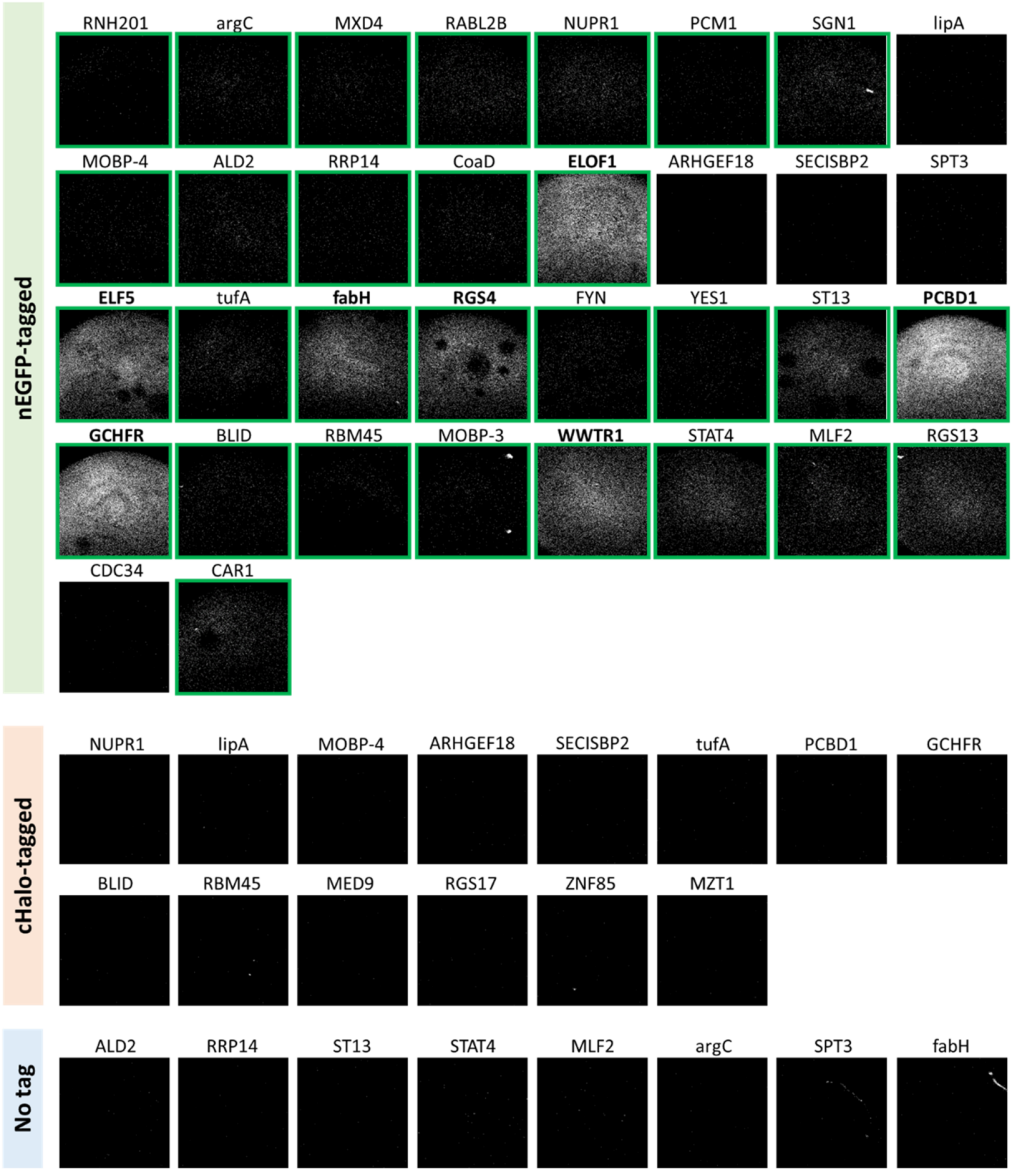
High-throughput screening for in vivo crystals. SONICC images of Sf9 cells expressing the indicated target proteins at 48 h p.i. Among nEGFP-tagged proteins, 29 targets were identified as positive hits as shown in green boxes, and 7 of them showed very strong SONICC signals with their names shown in bold. No signal was observed for proteins with cHalo tag or those without a tag.

To test whether the pipeline could identify any novel protein crystal targets, we expanded the SONICC detection to a large set of recombinant proteins for HT screening of in vivo microcrystals. A test collection of 34 proteins with nEGFP tag, 14 with cHalo tag, and 8 without a tag were produced and Sf9 cells expressing these recombinant proteins were imaged and analyzed by SONICC at 48 h p.i. (Fig. 6, Supplementary Table S4). Positive SONICC signals were observed for 29 proteins (~ 52% of all tested protein targets), and among them, 7 targets showed extremely strong signals (Fig. 6). Thus, SONICC screening results may indicate the presence of crystalline particles grown in the living insect cells, which can be potentially used for further X-ray diffraction studies.

**Figure 7:**
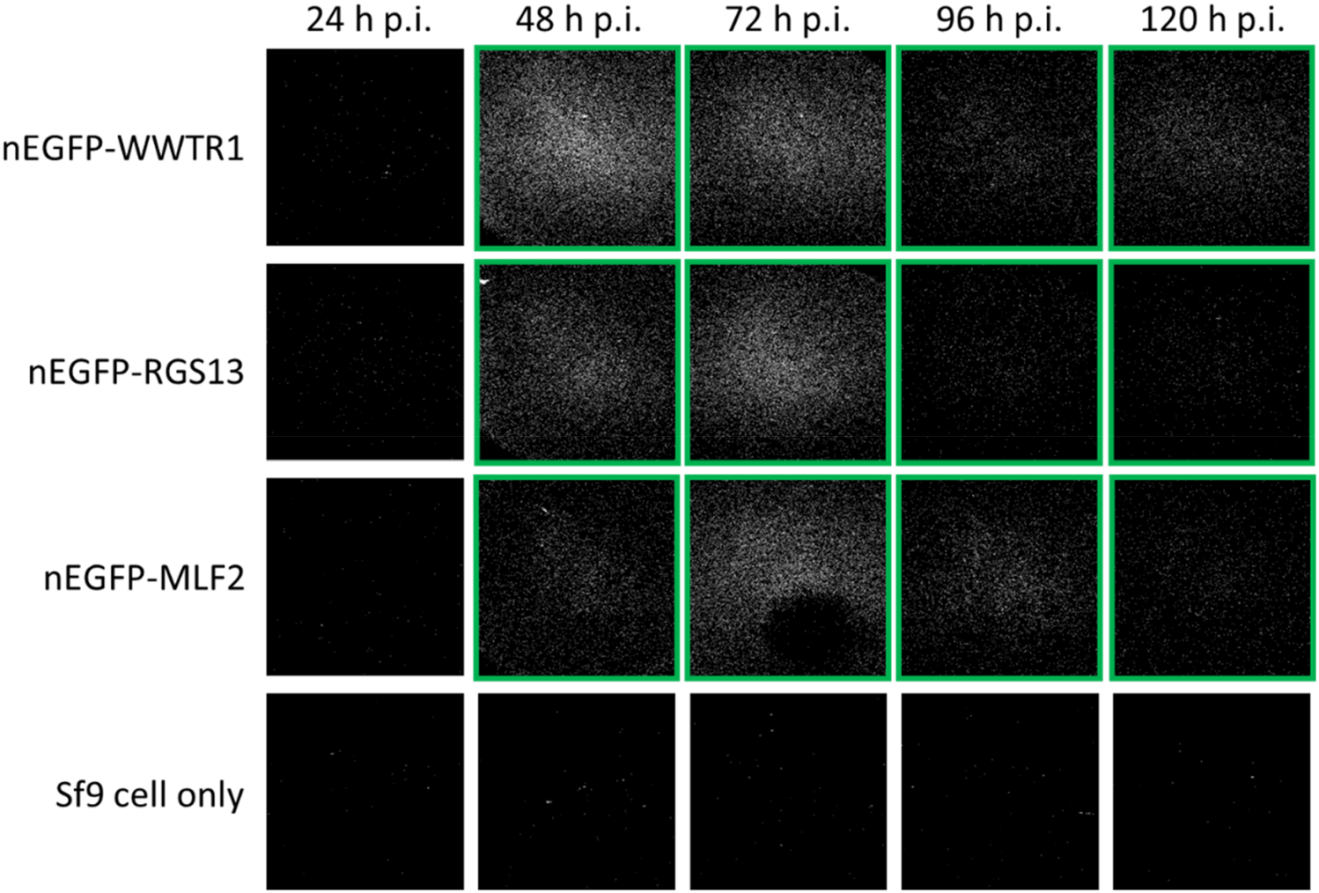
Time course of in vivo crystal formation. SONICC images of Sf9 cells expressing the indicated recombinant proteins or non-infected cells at 24, 48, 72, 96, and 120 h p.i. Targets that showed positive SONICC signals were shown in green boxes.

Furthermore, we monitored the time-dependent changes in protein crystallization by assessing the SONICC signals of target-expressing Sf9 cells harvested at different time points post infection. Three target proteins, MLF2, WWTR1, and RGS13, were expressed with the nEGFP tag, and were inspected by SONICC for the *in cellulo* crystallization signals over time. None of the tested target proteins exhibited positive SONICC signals before or at 24 h p.i. (Fig. 7). A strong signal was first observed for nEGFP-WWTR1 at 48 h p.i., which slowly dropped over time until no signals could be detected anymore after 120 h p.i. The first moderate signal was observed for nEGFP-RGS13 at 48 h p.i., followed by an increase to the maximal intensity at 72 h p.i., and then the signal declined rapidly afterwards. By comparison, nEGFP-MLF2 showed a mild signal at 48 h p.i., which then increased to the maximal intensity at 72 h p.i., followed by a slight decrease afterwards. These results may represent the protein-dependent variations in the growth dynamics of in vivo microcrystals. We hypothesize that crystals require a critical protein concentration in the cell for crystal formation, which is reached for most proteins after 48 h p.i. Crystal growth continues and maximizes after 72 h p.i. The decline of the crystals follows the decrease in cell viability. In addition, nEGFP-MLF2 and nEGFP-WWTR1 proteins displayed a punctate fluorescence pattern in a particular region of cells whereas nEGFP-RGS13 features an unevenly diffuse fluorescence in cells (Supplementary Fig. S7). Such differentiated fluorescence patterns might be associated with the difference in protein localization.

### Confirmation of in vivo crystallization by TEM imaging

**Figure 8:**
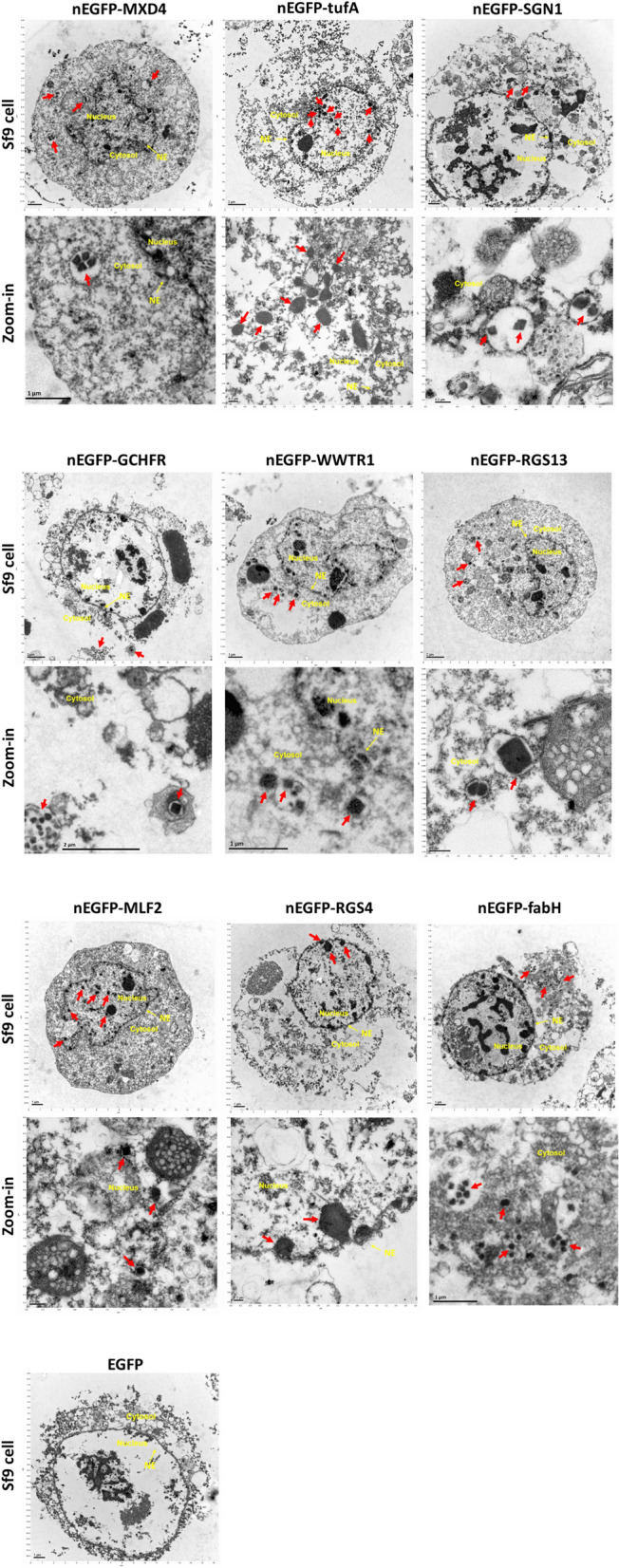
TEM of in vivo nanocrystals. Sf9 cells expressing the indicated SONICC-positive hits were examined by TEM to confirm the in vivo crystal formation. The lower panels include the zoom-in view of the Sf9 cells shown in the upper panel for each target. Nano- or micro-crystals of different cellular localization, size, and morphology were observed for each target, as indicated by the red arrows. EGFP was included as a negative control, and no identifiable crystals were observed inside the cells. Cell compartments (cytosol, nucleus, and nuclear envelope (NE)) have been highlighted for clarity in all images.

To further confirm the crystal formation in insect cells, a total of 18 SONICC-positive hits were further examined using TEM technique. The presence of nano- or micro-crystals characterized by sharp edges was identified for MXD4, tufA, SGN1, GCHFR, WWTR1, RGS13, MLF2, RGS4, and fabH samples (Fig. 8), which is in agreement with the SONICC screening results. The presence of non-sharp particles was also observed in the rest of the samples, making the crystallinity of these targets less conclusive (Supplementary Fig. S6). Sf9 cells expressing EGFP alone were included as the negative control, and in contrast, no identifiable crystalline particles were seen in these cells with TEM (Fig. 8).

Distinct morphology, size, and cellular localization were observed for each target (Fig. 8). Crystalline particles appeared hexagonal (e.g. tufA, RGS4), cubic (e.g. RGS13, MLF2), or quadrilateral (e.g. SGN1) structures. They were located in either cytosol (e.g. MXD4, SGN1, fabH), nucleus (e.g. tufA), or both (e.g. MLF2). It was also noticed that the nanocrystals for some targets were enclosed in cytoplasmic vesicles with either single (e.g. MLF2) or multiple (e.g. MXD4, RGS13) crystalline particles inside. The number of crystals per cells and the portion of cells containing crystals varied by targets.

## Discussion

In this study, we have established a BEVS-based protein production pipeline to enable mass parallel recombinant protein expression and rapid screening for in vivo microcrystals. We took advantage of the pIEx/BacMagic-3 expression vector system and Gateway cloning technology, and constructed a suite of pIEx-based expression vectors that support: (1) convenient HT construction of expression clones by single-step Gateway recombinational cloning; and (2) mass parallel expression of target proteins with different fusion tags of choice at either the N- or C-terminus to meet various protein research needs. We first demonstrated the successful application of our new vectors for overexpression of proteins in Sf9 cells (Fig. 4, Supplementary Fig. S2) and their subsequent protein purification (Fig. 5). We further demonstrated that the new system can be used for in vivo crystallization screening prior to scaled-up production of microcrystals for structural and functional studies using a set of target proteins that vary in their host organism, molecular weight, and subcellular localization (Fig. 6, Supplementary Table S1).

Insect cells were chosen as the primary expression system for this pipeline because of their relatively high protein production level and broad eukaryotic protein processing abilities^17^. In vivo protein crystallization has been observed in nature in all kingdoms of life, where it is often functionally associated with protein storage, protection, and stabilization^5,37^. While native crystal formation usually provides advantageous functions for the organism, some accidental intracellular crystallization, induced by small environmental or structural changes, is considered to be related to certain diseases^5,6^. For example, Charcot-Leyden crystals (CLCs)^38,39^, which are spontaneously formed through auto-assembly of galectin-10 protein in lymphocytes at diverse human tissues and body fluids, are involved in various allergic, parasitic, neoplastic and inflammatory disorders^40,41^. However, the role of CLCs in lymphocytes still remains obscure^41^. Other than the native crystal formation, the crystalline state has also been observed as a consequence of heterologous protein expression in host cells, which may be associated with the high local concentration of active protein. Driven by this phenomenon, several additional examples of crystals has been discovered in living cells, including bacteria^42^, plant cells^43,44^, mammalian cells^45,46^, as well as baculovirus-infected insect cells^7,47^.

The applicability of in vivo crystals in structural biology was first established at a synchrotron radiation source. In 2007, the first structure of a natively crystallizing protein, cypoviral polyhedra, was reported by Coulibaly and coworkers using the synchrotron diffraction approach^48^. Based on this success, this approach was subsequently applied to the structural characterization of baculoviral polyhedra protein using in vivo-grown microcrystals purified from insect cells, resulting in the determination of the 2.2 Å resolution structure^49^. These proof-of-concept studies elucidated that *in cellulo*-grown crystals can be used as suitable targets to extract high-resolution structural information. However, in contrast to countless efforts devoted to in vitro crystallization over the past decades, the potential of in vivo crystals in structural biology has not yet been fully valued and exploited, mainly because the relatively small microcrystals formed in vivo are sensitive to radiation damage caused by conventional synchrotron X-ray radiation sources^46^. Fortunately, this challenge has been recently overcome by the emerging SFX technique, which uses extremely bright and ultrashort X-ray pulses from XFEL sources to irradiate nano- or micro-crystals and record their diffraction before destruction occurs, therefore boosting the applicability of in vivo crystallization in structural biology^9,46^. The first study reporting the successful synergy of in vivo crystallization and SFX technology was published in 2013 by Redecke and co-workers^11^, in which they revealed the 2.1 Å resolution structure of TbCatB protein. This breakthrough experiment demonstrated that structural information can be obtained by the “diffraction-before-destruction” approach of SFX from thousands of microcrystals that are delivered to the XFEL beam in a liquid jet in their mother liquor^46^.

The feasibility of solving protein structures using in vivo-grown microcrystals entails a HT pipeline to optimize protein expression, in vivo crystal formation and structure determination protocols. We included nEGFP-μNS in our study as a positive control for in vivo crystallization and achieved high expression and good crystal formation (Supplementary Fig. S5). Similar results were observed in repeated experiments, suggesting such crystallization in living insect cells is highly reproducible. During the course of infection, the number of crystals continuously increased until most cells contained one or multiple crystals bundled together. The size of nEGFP-μNS crystals generally did not exceed the living insect cell dimensions (~ 15–20 µm) (Supplementary Fig. S5A). Interestingly, no obvious degradation of nEGFP-μNS crystals was observed after crystal-containing cells were stored at 4 °C for up to 2 weeks, although some crystal particles were seen floating freely in the medium or attached to cell remnants (Supplementary Fig. S5C). This indicates the good intrinsic stability of nEGFP-μNS crystals in agreement with the previous work by Schönherr et al.^8^. We further used these nEGFP-μNS crystals successfully in SFX diffraction experiments being able to build the first electron density maps of the nEGFP-μNS protein^36^, which demonstrates the feasibility of using in vivo-grown crystals produced from our pipeline for structural studies.

The rapid HT *in cellulo* crystal screening built on the current pipeline largely accelerates the identification of protein candidates that can form microcrystals prior to proceeding with in-depth structural characterization by SFX. Here, apart from µNS, positive SONICC signals were detected for 29 out of the 56 recombinant proteins tested (Fig. 6), suggesting that when proteins are overexpressed in insect cells, the formation of microcrystals might be a more frequent event than previously known^5,6^. Interestingly, all positive hits identified in this study were nEGFP-tagged proteins, while no SONICC signals were observed for any target proteins expressed without tags or with the cHalo tag (Fig. 6). Thereby, EGFP seems to be an efficient tag in promoting the crystallization of fusion proteins and enhancing microcrystal detection^8^.

The in vivo crystallization of 18 SONICC-positive hits was further verified by TEM, and 9 targets were confirmed positive for nanocrystals characterized by sharp edges (Fig. 8), in either the nucleus, the cytoplasm, or both. Although non-sharp particles were also observed in the other 9 targets (Supplementary Fig. S6), the absence of sharp edges poses uncertainty in their crystallinity. To address this issue, further confirmation by diffractive techniques is required to provide definitive evidence. Nevertheless, the demonstration of frequent in vivo crystallization remains a promising result for future studies.

As evidenced by TEM, the nanocrystals displayed distinct morphology, such as hexagonal, cubic, or quadrilateral structures (Fig. 8). Nanocrystals of some targets were enclosed in cytoplasmic vesicles with either single or multiple crystalline particles inside. More characterization of expanded in vivo crystallized proteins might illuminate the association of these parameters to the crystallization process. It is important to note that all 9 samples were inspected by X-ray powder diffraction using an X-ray home source. As expected, the flux of the home source was too weak to observe the powder diffraction rings, even for the μNS crystals that diffracted well at an XFEL. The presence of such tiny crystals observed by TEM clearly indicates that more powerful X-ray radiation sources such as XFELs would be indeed required to observe diffraction from such small crystals.

Further X-ray diffraction of isolated crystals by SFX can be performed in the future to confirm the results and potentially determine the protein structures at high resolution; however, experimental “beamtime” at XFELs is quite limited as only 5 XFELs exist so far worldwide, and only one experiment can be performed at a given time. Microfocus beamlines at synchrotrons with high flux and μm focus have also been recently used for successful structure determination of larger in vivo-grown crystals using serial millisecond X-ray crystallography (SMX). With even more SMX synchrotron beamlines under development and new developments in compact XFEL technology at Arizona State University^50^ and in Germany at DESY^51^, in vivo-grown crystals may soon become a commonly used route for protein crystallography.

Time-dependent monitoring of SONICC signals of three nEGFP-tagged proteins revealed that microcrystal formation of different target proteins followed various temporal patterns in terms of signal intensity and duration, which further emphasizes the importance of a HT pipeline to optimize the conditions for growth of structure-grade in vivo microcrystals. The nEGFP-WWTR1 protein showed a strong signal early at 48 h p.i., which slightly declined over time and lasted for up to 120 h p.i. The nEGFP-MLF2 and nEGFP-RGS13 proteins developed a mild-to-moderate signal around 48 h p.i., which subsequently reached a peak at 72 h p.i. and then decreased at different rate (Fig. 7). The intracellular crystallization process is highly dynamic as reported in the case of firefly luciferase crystals, which display a growth period of dynamic degradation and re-assembly^8^. Such protein-dependent variations in crystallization dynamics may indicate that crystal growth and degradation kinetics may vary for different protein targets depending on their expression level and subcellular localization^8^. In addition, different fluorescence patterns were observed within Sf9 cells expressing these target proteins (Supplementary Fig. S7), although it is not yet clear how such difference relates to the in vivo crystallization process. In the future, further proteomic-scale investigation on a larger set of target proteins could provide even more insights to how the time-dependent crystal formation and/or fluorescence morphology correlate with promising X-ray diffraction results.

Out of 156 expression clones tested in this study, 120 were expressed, as verified by Coomassie-stained SDS-PAGE gels, representing an expression success rate of ~ 77% (Supplementary Table S2). Small-scale protein purification of 48 recombinant proteins showed that 50% of targets were soluble and could be purified (Supplementary Table S3). Furthermore, SONICC screening in conjunction with TEM imaging identified the presence of in vivo crystals for 9 targets out of 56 recombinant proteins, suggesting a crystallization rate of ~ 16% (Supplementary Table S4). These numbers are comparable to the data from other HT structural genomics platforms according to the Protein Structure Initiative Structural Genomics Knowledgebase (PSI SGKB, https://kb.psi-structuralgenomics.org)^52^. The portions of targets failing at each step of expression, purification, and crystallization for structure studies are ~ 10%, ~ 62%, ~ 31% in the Joint Center for Structural Genomics (JCSG), ~ 16%, ~ 57%, ~ 49% in the Northeast Structural Genomics Consortium (NESG), and ~ 77%, ~ 29%, ~ 35% in the New York Structural Genomics Research Center (NYSGRC), respectively. Given that the purification screening was only performed on a subset of all expressed targets in our study and that certain proteins might be difficult to be purified, in the future, an expanded investigation involving more protein targets could reveal a more detailed and comprehensive landscape of protein solubility and crystallizability for targets expressed from our pipeline. The incorporation of Gateway cloning technology into the pIEx/BacMagic-3 system not only makes the pipeline amenable for HT construction of expression clones but also leverages the readily available ORF libraries for functional proteomics^53–55^. For example, our DNASU plasmid repository comprises ORFs encoding the proteomes for human, yeast, *Drosophila*, *Arabidopsis*, *Xenopus*, and hundreds of different bacteria and viruses. This provides plentiful starting materials to satisfy researchers’ needs to study their proteins of interest.

In summary, we have developed a versatile baculovirus-mediated expression pipeline by constructing a suite of Gateway-compatible pIEx expression vectors with various fusion tags, which enables HT protein expression and in vivo crystallization for functional and structural studies. In conjunction with the existing Gateway clone libraries and the advancement of XFEL technology, these vectors will enable proteomic-scale optimization of protocols for structure determination using in vivo microcrystals.

## Methods

### Construction of pIEx expression vectors

The Gateway-compatible pIEx expression vector series were modified from the pIEx-cyto vector (obtained as a gift from Drs. James Love and Scott Garforth at Albert Einstein College of Medicine; available in DNASU) as illustrated in Fig. 2. Briefly, the cloning efforts were structured in two steps, with the pIEx-nGST expression vector shown as an example here. To generate pIEx-nGST expression vector, the pIEx-cyto vector was first digested with NcoI and Bsu36I, followed by gel purification to remove the original insert. In parallel, the GST tag coding sequence was amplified from pANT7-cGST vector (available in DNASU). The purified PCR product of GST tag was inserted to the linearized pIEx-cyto vector via In-Fusion reaction, to generate the pIEx-nGST empty vector. The In-Fusion product was transformed to *E. coli* DH5α competent cells (NEB) for colony selection. Following the plasmid isolation and sequence verification, the pIEx-nGST empty vector was digested with SgfI and Bsu36I, followed by gel purification to remove the original insert. In parallel, the Gateway death cassette was amplified from a modified pANT7-cGST-DC vector (available in DNASU). The purified PCR product of the death cassette was inserted into the linearized pIEx-nGST empty vector via In-Fusion reaction, to generate pIEx-nGST expression vector. The final In-Fusion product was transformed to *E. coli ccdB* survival-competent cells (Invitrogen) for colony selection.

The resulting pIEx expression vectors were sequence verified for the presence of both the death cassette and fusion tags, which enable the insertion of target gene through Gateway LR reaction as well as the production of N- or C-terminally tagged fusion proteins through homologous recombination with BacMagic-3 DNA. Maps and sequences of these Gateway-compatible pIEx expression vectors are available in DNASU (https://dnasu.org/DNASU/). The plasmid DNA of all expression vectors was prepared using the NucleoBond Plasmid Maxiprep Kit (Macherey-Nagel).

### Protein selection

A test collection of 40 full-length proteins were selected to assess the expression capability of our pipeline (Supplementary Table S1). These proteins were chosen if their full-length ORFs were available in a Gateway donor clone in DNASU. The selected proteins (1) range in size from ~ 8 to 130 kDa, (2) localize in different subcellular compartments, and (3) function in diverse biological processes. The donor clones for selected ORFs in the test collection were acquired from DNASU to construct the pIEx expression clones for protein expression with various tags in insect cells. These ORFs were annotated as either “closed” or “fusion” to indicate if a stop codon is present (closed) or is absent (fusion) in an ORF insert. Fusion format clones are used for producing a C-terminally tagged version of an ORF.

### Gateway subcloning of pIEx expression clones

The Gateway LR cloning was performed to construct multiple pIEx expression clones in parallel. The LR cloning reaction was set up by mixing 300 ng of Gateway donor clone, 300 ng of pIEx expression vector, 1 μL of Gateway LR Clonase II Enzyme Mix (Invitrogen), and then incubated for 1 h at 25 °C (Fig. 3A). The cloning reaction mix was transformed into 20 μL of *E. coli* DH5α competent cells and incubated in 150 μL of S.O.C medium (Thermo Scientific) for 1 h at 37 °C, 250 rpm in an orbital shaker. The entire cell suspension was plated on lysogeny broth (LB) agar with 100 μg/mL of ampicillin followed by an overnight incubation at 37 °C. Positive colonies selected from the agar plates were inoculated to LB medium with 100 μg/mL of ampicillin. The plasmid DNA of the resulting pIEx expression clones was isolated from the bacteria culture using the NucleoSpin Plasmid Miniprep Kit (Macherey-Nagel). All LR cloning products were sequence verified prior to transfection of insect cells.

### Insect cell culture

*Spodoptera frugiperda* Sf9 cells (Invitrogen) were maintained in Sf-900 III Serum Free Medium (Gibco) and incubated at 27 °C, 140 rpm without CO_2_ exchange in a non-humidified orbital shaker. Suspension culture was passaged when reaching a density of 2E6 viable cells/mL and was seeded at 0.5E6 viable cells/mL. Cell counting was performed on the suspension culture using Trypan Blue (Invitrogen) to determine the cell density and viability at every passage.

### Recombinant baculovirus generation and amplification in 24-well plate format

The pIEx/BacMagic-3 co-transfection was performed for multiple expression clones to generate recombinant baculovirus in parallel. For each transfection, a reaction was assembled by mixing 1 mL of Sf-900 III Serum Free Medium, 5 μL of Insect GeneJuice Transfection Reagent (Sigma-Aldrich), 100 ng of BacMagic-3 DNA (Novagen), and 500 ng of pIEx expression clone (Fig. 3B). The transfection reaction was gently agitated and incubated at room temperature (RT) for 30 min to allow complexes to form. The entire reaction was then slowly added to 1 mL of Sf9 cells at 1E6/mL in a 24-well deepwell plate (Thomson Instrument Company) for recombinant virus production. Plates were sealed with an adhesive silicone film (Analytical Sales & Services) to allow air exchange, and crucially, to avert evaporation. Cultures were incubated for 120 h at 27 °C, 140 rpm. Subsequently, the culture was centrifuged at 1,000 × *g* for 5 min to remove cell debris and the supernatant containing recombinant budded viruses was harvested. The resulting first generation (P1) of virus was then amplified through a second round of Sf9 cell infection to generate P2 virus. Briefly, 4 mL of Sf9 cells at 2E6/mL were infected with 20 μL of P1 virus stock in a 24-well deepwell plate, sealed with silicone film, and incubated for 120 h at 27 °C, 140 rpm. The generated P2 virus was verified via the expression screening for the working stock prior to protein expression.

### Protein expression in 24-well plate format

In a 24-well deepwell plate, 4 mL of Sf9 cells at 1E6/mL were infected with 20 μL of P2 virus stock which had been confirmed for protein expression, and incubated for 72 h at 27 °C, 140 rpm. Alternatively, suspension culture was scaled up in a sterile Optimum Growth Flask (Thomson Instrument Company) by adding P2 virus stock proportionally with the same virus-to-cell ratio. After centrifugation of infected culture, the insect cell pellet was collected for SDS-PAGE analysis on 4–20% precast polyacrylamide gels (Bio-Rad). Gels were stained with Coomassie SimplyBlue SafeStain (Invitrogen) to visualize the protein bands. A target was considered as “expressed” if a novel band at expected size was present only in infected cells but not in uninfected cells.

### Protein purification

One tablet of Protease Inhibitor Cocktail (Roche), 500 μL of Insect Popculture (Millipore), and 1.6 μL of Benzonase (Millipore) were added to 10 mL of Sf9 cell culture expressing the target protein and incubated for 15 min at RT. The resulting lysate was subjected to purification using different types of affinity beads, depending on the fusion tag.

For His-tagged proteins, 400 μL of 50% Ni-NTA agarose (QIAGEN) was washed and equilibrated with 1 mL of equilibration buffer (50 mM Tris, 300 mM NaCl, 1 mM DTT, 5% (v/v) glycerol, 1% (v/v) Triton X-100, pH 7.5), and incubated with 10 mL of total lysate for 1 h at 4 °C with agitation. Agarose beads were then washed twice subsequentially with 10 mL of wash buffer 1 and 2 (20 mM and 50 mM imidazole in equilibration buffer) to remove unbound particles, and then were incubated with 500 μL of elution buffer (250 mM imidazole in equilibration buffer) for 5 min at RT with agitation to elute the His-tagged protein targets.

For GST-tagged proteins, 100 μL of 25% Glutathione Magnetic Agarose Beads (Thermo Scientific) were washed twice with 500 μL of wash buffer (125 mM Tris, 150 mM NaCl, 1 mM DTT, 1 mM EDTA, pH 7.4). Lysate was centrifuged at 2,000 × *g* for 5 min at 4 °C and the resulting supernatant was incubated with agarose beads for 1 h at RT with agitation. Agarose beads were washed twice with 500 μL of wash buffer to remove unbound particles, and then were incubated with 250 μL of elution buffer (50 mM reduced glutathione in wash buffer) for 10 min at RT with agitation to elute the GST-tagged protein targets.

For the small-scale protein purification, 4 mL of Sf9 cell cultures expressing the target proteins with different fusion tags were harvested and lysed. The resulting cell extracts were incubated with appropriate commercial magnetic beads according to the manufacturer’s instructions. Specifically, GFP-Trap_MA beads (Chromotek) were used to purify nEGFP- and cGFP-Avi-His-tagged proteins; MagneHaloTag beads (Promega) for cHalo- and NcoI-cHalo-tagged proteins; Dynabeads His-Tag beads (Thermo Scientific) for nHis- and cHis-tagged proteins; MagneGST beads (Promega) for nGST-tagged proteins; anti-FLAG-M2 Magnetic beads (Sigma-Aldrich) for nHAsq-FLAG-His-tagged proteins. The resulting purified fractions were analyzed by Western blot using appropriate anti-fusion tag primary and secondary antibodies. Specifically, GFP (3E6) Mouse mAb (Invitrogen) was used to purify nEGFP- and cGFP-Avi-His-tagged proteins; HaloTag Rabbit pAb (Promega) for cHalo- and NcoI-cHalo-tagged proteins; His-Tag (27E8) Mouse mAb (Cell Signaling Technologies) for nHis- and cHis-tagged proteins; GST (26H1) Mouse mAb (Cell Signaling Technologies) for nGST-tagged proteins; FLAG Rabbit pAb (Sigma-Aldrich) for nHAsq-FLAG-His-tagged proteins. HRP-linked anti-rabbit or anti-mouse IgG antibodies (Cell Signaling Technologies) were used for detection of target bands.

### SONICC screening for microcrystals within living Sf9 cells

Sf9 cells infected with recombinant virus were tested for *in cellulo* crystallization using a SONICC instrument. Briefly, at 24, 48, 72, 96, and 120 h p.i., 1 mL of each suspension culture was harvested and centrifuged at 500 × *g* for 5 min at 4 °C. The supernatant containing the culture medium was discarded, and the insect cell pellet was gently re-suspended in 50 μL of PBS (phosphate-buffered saline) buffer. Next, 2 μL of high-density cell suspension was loaded into a 96-well 2-drop MRC Crystallization Plate (Swissci) and immediately imaged with SONICC imager (Formulatrix) using visible light and the SHG technology to visualize and identify the in vivo-grown protein crystals. In a typical SONICC image, crystals appear white against a stark black background that helps to identify crystals even in murky environments like those from the extremely complex and crowded cellular environments. Image tuning was utilized to adjust the brightness and contrast of the SONICC images to remove any noise visible from the drops of each target. In our experiment, the signal intensity of the images was auto tuned per drop by default settings in Rock Maker (Formulatrix) and compared against the control drop containing the non-infected Sf9 cells to determine the positive hits.

### TEM imaging of SONICC-positive hits

TEM experiments were performed to examine the intracellular location, size and morphology of crystals of 18 protein targets (PCBD1, RBM45, BLID, MXD4, ELF5, tufA, SGN1, RABL2B, NUPR1, ELOF1, fabH, GCHFR, WWTR1, STAT4, RGS13, MLF2, CAR1 and RGS4). The EGFP sample was included as negative control. Recombinant protein targets were expressed and crystallized in Sf9 insect cells as described above. Cells were prepared using standard TEM fixation protocol with modifications^56–58^. Infected insect cells were fixed using 2% (v/v) glutaraldehyde in fresh cell growth buffer (Sf-900 III Serum Free Medium) for 15 min at RT and incubated on ice for 2 h for primary fixation. Cells were subsequently washed four times (10 min each) using the cells growth buffer and stored overnight in the same buffer. Next, cells were subjected to a secondary fixation step in 1% (w/v) osmium tetroxide in PBS buffer for 2 h on ice followed by staining in 0.5% (w/v) uranyl acetate (UA) overnight at 4 °C. Excess UA was removed by washing four times (10 min each) with deionized water (diH_2_O). Complete dehydration in acetone was followed by infiltration and embedding in Spurr’s epoxy resin. Using the standard procedures for sectioning, 70 nm sections were cut using a Leica Ultracut-R microtome and collected on formvar-coated copper slot grids followed by post-staining using 2% UA in 50% ethanol and Sato’s lead citrate. Imaging was performed using a Philips CM 12 TEM instrument and images were collected on a Gatan model 791 side-mount CCD camera.

## Supplementary information

Supplementary Figures

Supplementary Table S1.

Supplementary Table S2.

Supplementary Table S3.

Supplementary Table S4.
