# Supplementary material for: Construction of gateway-compatible baculovirus expression vectors for high-throughput protein expression and in vivo microcrystal screening
Source: Sci Rep. 2020 Aug 7;10:13323. doi: 10.1038/s41598-020-70163-2 (PMC7414197; doi:10.1038/s41598-020-70163-2)
Supplement: Supplementary file 1 — Supplementary Figures [file 41598_2020_70163_MOESM1_ESM.docx]

**Construction of Gateway-compatible baculovirus expression vectors for high-throughput protein expression and *in vivo* microcrystal screening**

**Yanyang Tang^1,2^, Justin Saul^2^, Nirupa Nagaratnam^1,3^, Jose M. Martin-Garcia^3^, Petra Fromme^1,3^, Ji Qiu^2*^, Joshua LaBaer^1,2*^**

^1^School of Molecular Sciences, Arizona State University, Tempe, AZ, 85287, USA

^2^Virginia G. Piper Center for Personalized Diagnostics, The Biodesign Institute, Arizona State University, Tempe, AZ, 85281, USA

^3^Center for Applied Structural Discovery, The Biodesign Institute, Arizona State University, Tempe, AZ, 85281, USA

*Joshua LaBaer: Joshua.Labaer@asu.edu; Ji Qiu: Ji.Qiu@asu.edu

**
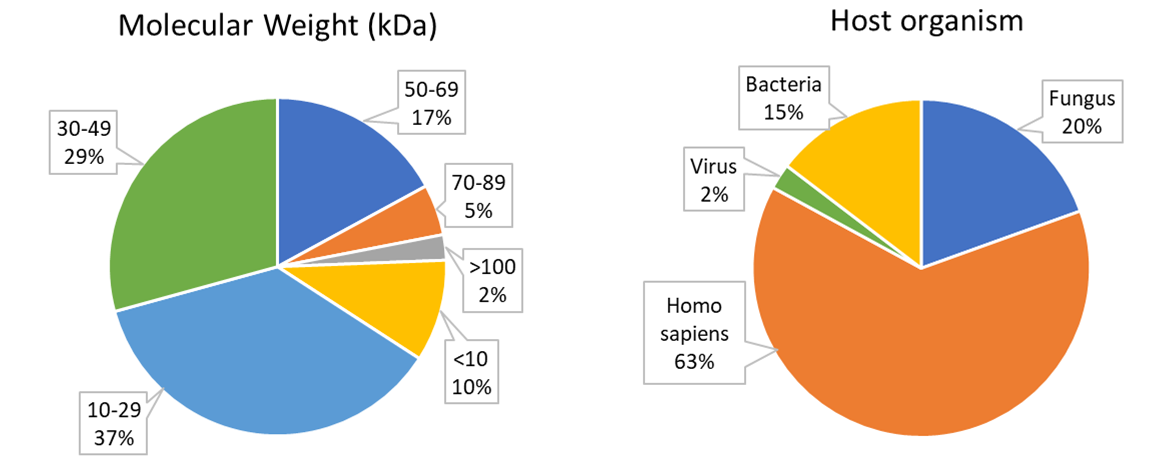
** **Supplementary Figure S1.** **Host organisms and molecular weight range of the test collection.** Proteins selected for the test collection are derived from a variety of species, including human, fungus, bacteria, and virus, with molecular weights ranging from ~8 to 130 kDa as indicated.

**
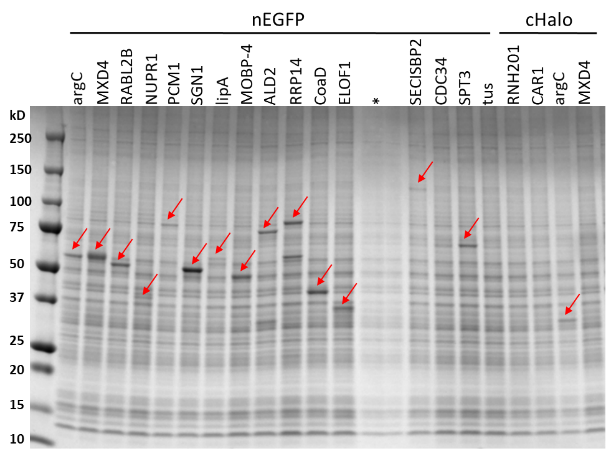
**


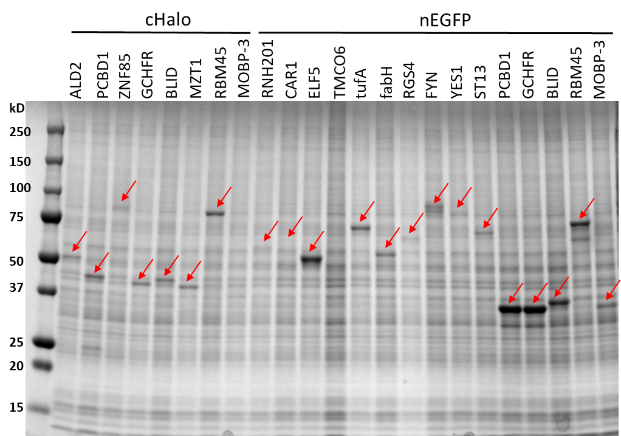


**
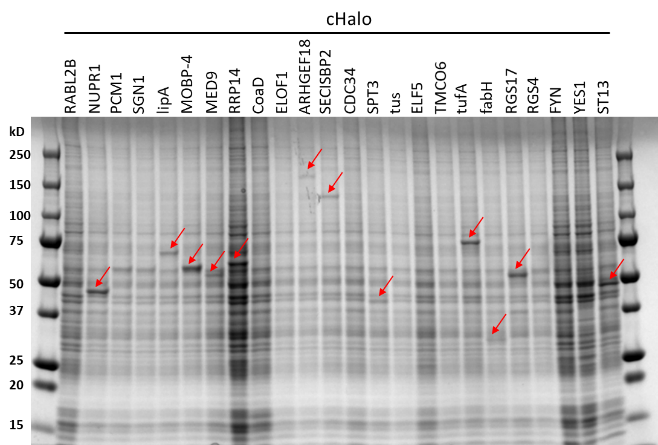
**

**
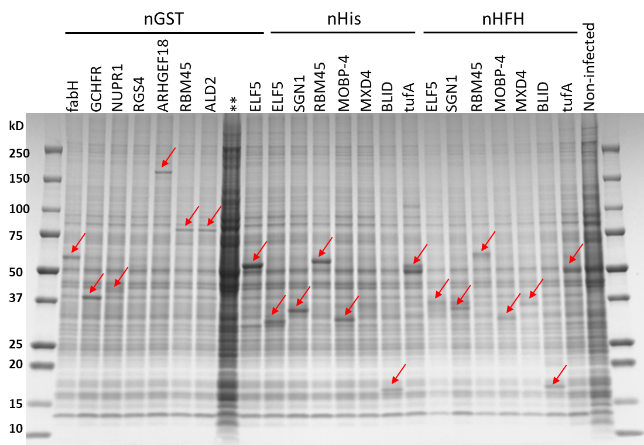
**

**
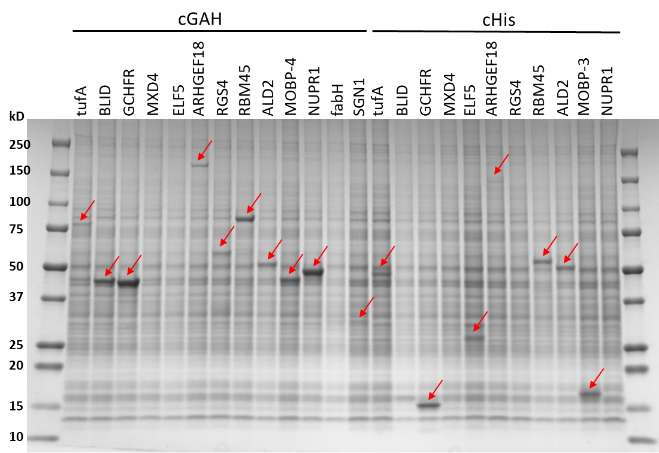
**

**
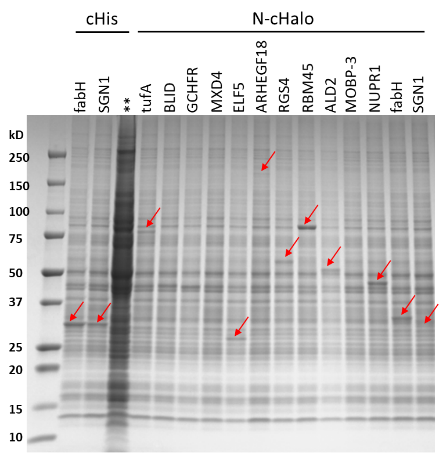
**


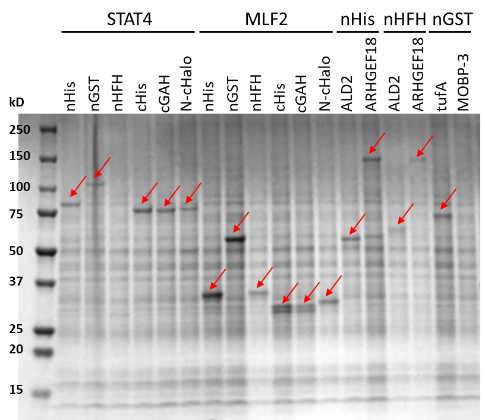


**Supplementary Figure S2. Successful mass parallel expression of recombinant proteins fused to various fusion tags.** Target proteins in the test collection were expressed using the indicated pIEx expression vectors and analyzed by Coomassie-stained SDS-PAGE. A target was considered as “expressed” if a novel band (red arrows) was present at the expected molecular weight but absent in the non-infected Sf9 cells. Closed format clones were attached with no fusion tags when expressed using pIEx vectors carrying C-terminal tags. * indicates an inter-lane sample diffusion due to the broken gel well; ** indicates a sample overloading.

**
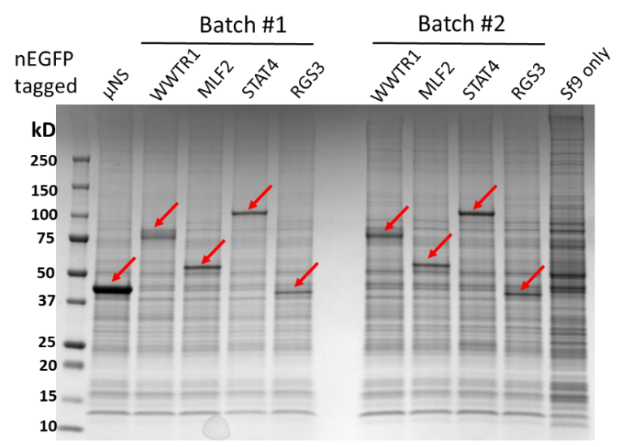
**

**Supplementary Figure S3. Reproducibility of protein expression.** The pIEx expression clones for the indicated targets were introduced into the pipeline repeatedly. Harvested cultures were analyzed by Coomassie-stained SDS-PAGE to assess the inter-batch variations in protein expression. In both batches, all proteins were overexpressed (red arrows) compared to uninfected Sf9 cells. The nEGFP-μNS was included as a positive control for expression.


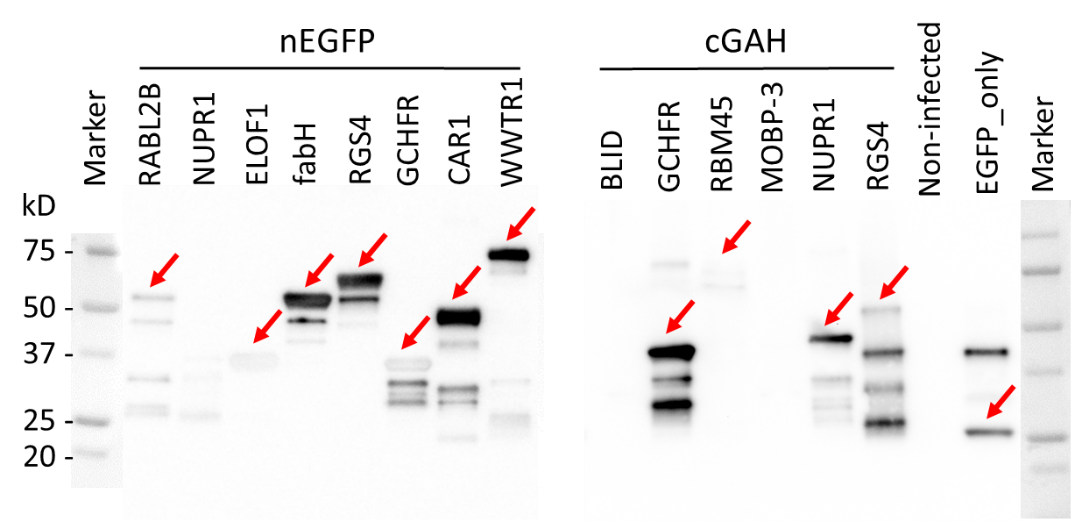


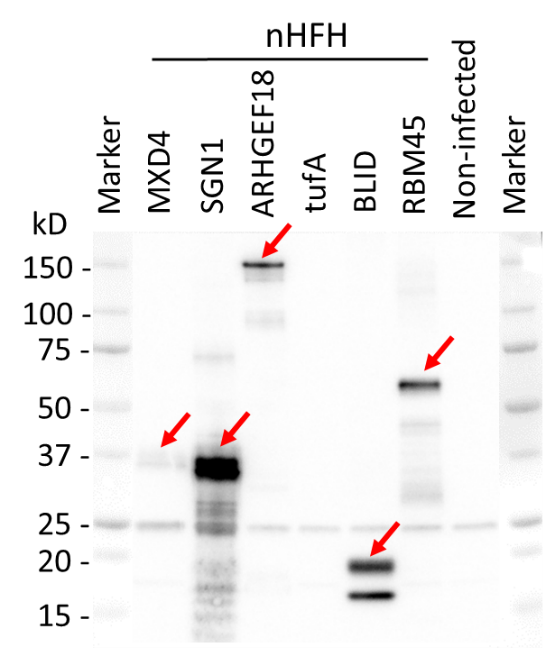

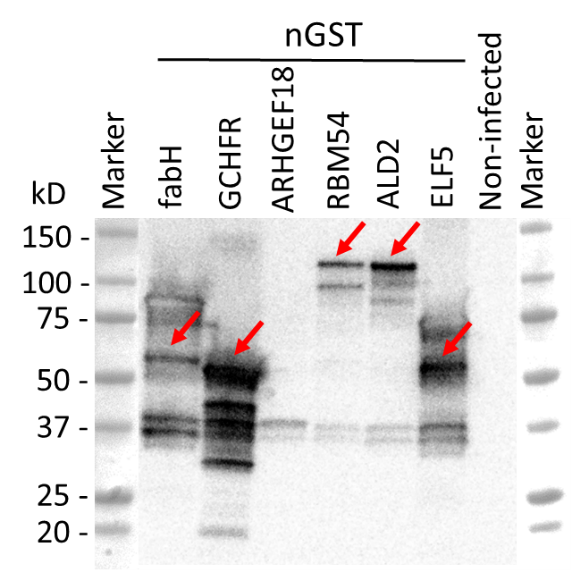


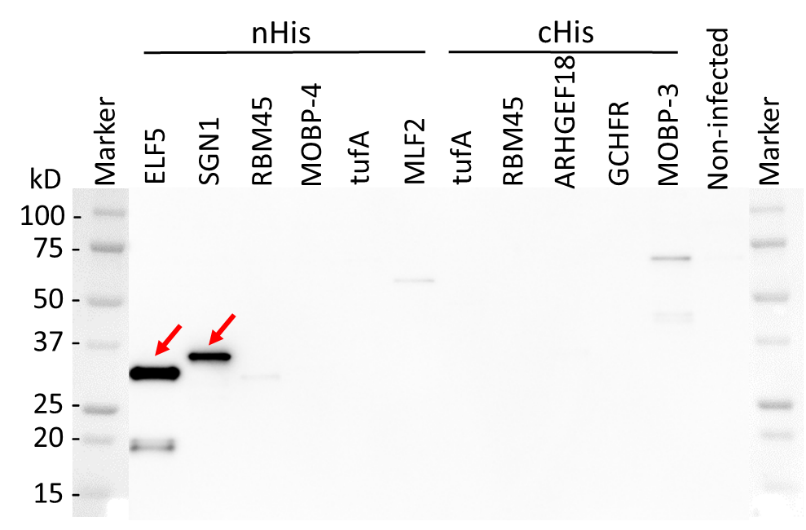


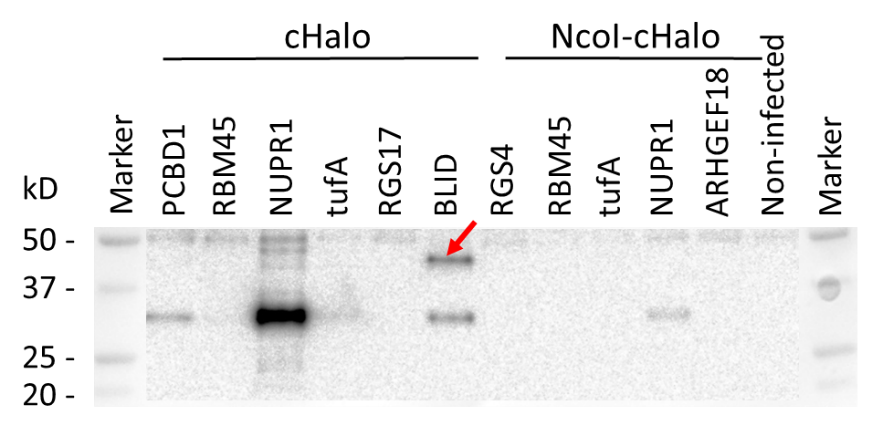


**Supplementary Figure S4. Small-scale protein purification of recombinant proteins.** Cell extracts expressing indicated recombinant proteins were subjected to pulldown assays using appropriate magnetic beads and detected by Western blot with anti-fusion tag antibodies. Targets that were successfully purified are indicated by red arrows.

**
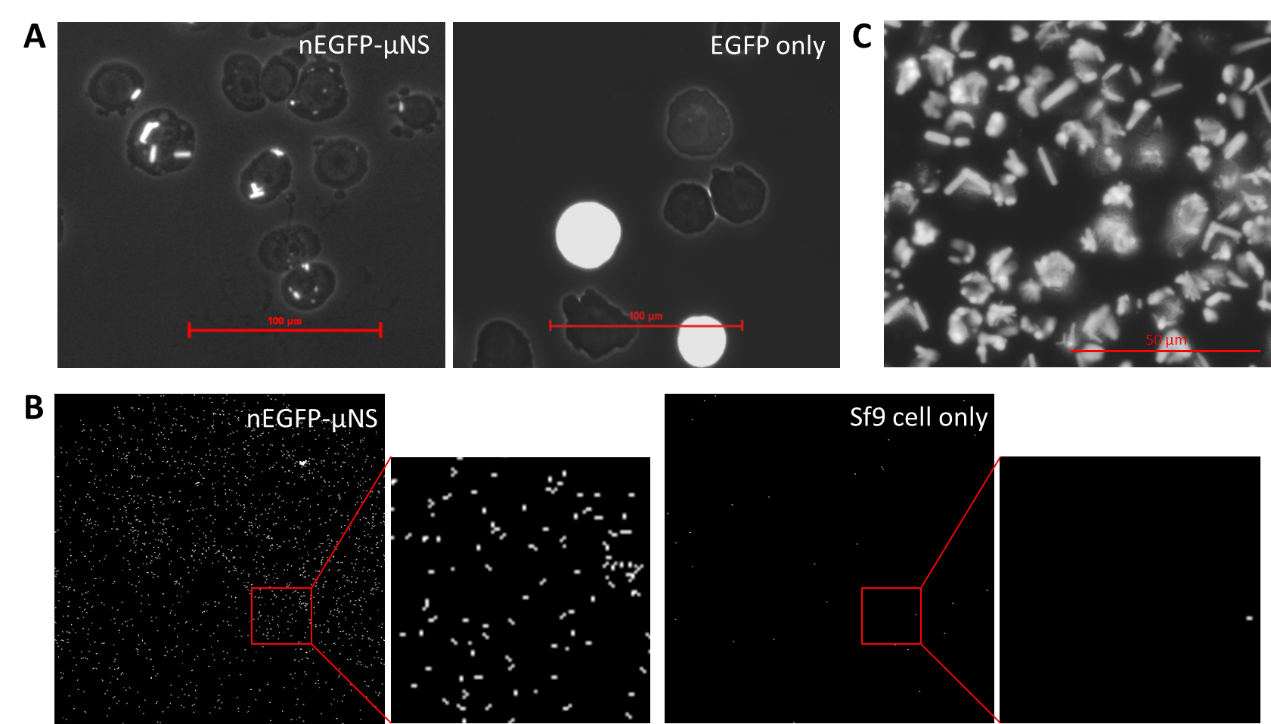
** **Supplementary Figure S5. Characterization of *in vivo* crystallization in Sf9 cells expressing nEGFP-μNS**. **A)** UV fluorescence images revealed one or more rod-shaped crystals in Sf9 cells expressing nEGFP-μNS but not in cells expressing EGFP tag alone. **B)** SONICC detected the positive signal for Sf9 cells infected with nEGFP-μNS while no signal for non-infected control. A zoom-in view was shown for the red boxed area. **C)** UV fluorescence image showed intact nEGFP-μNS crystal particles in suspension after the culture was stored at 4 °C for 2 weeks.


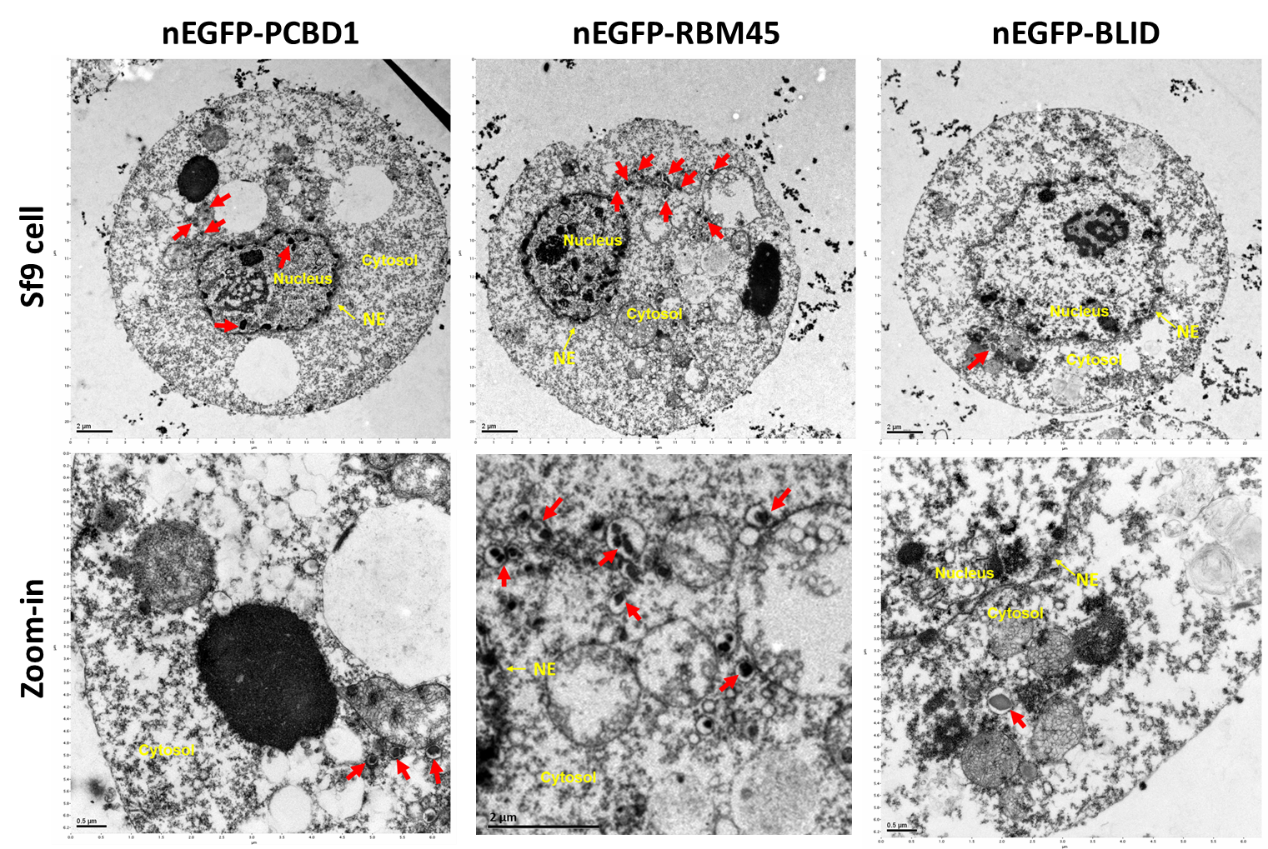


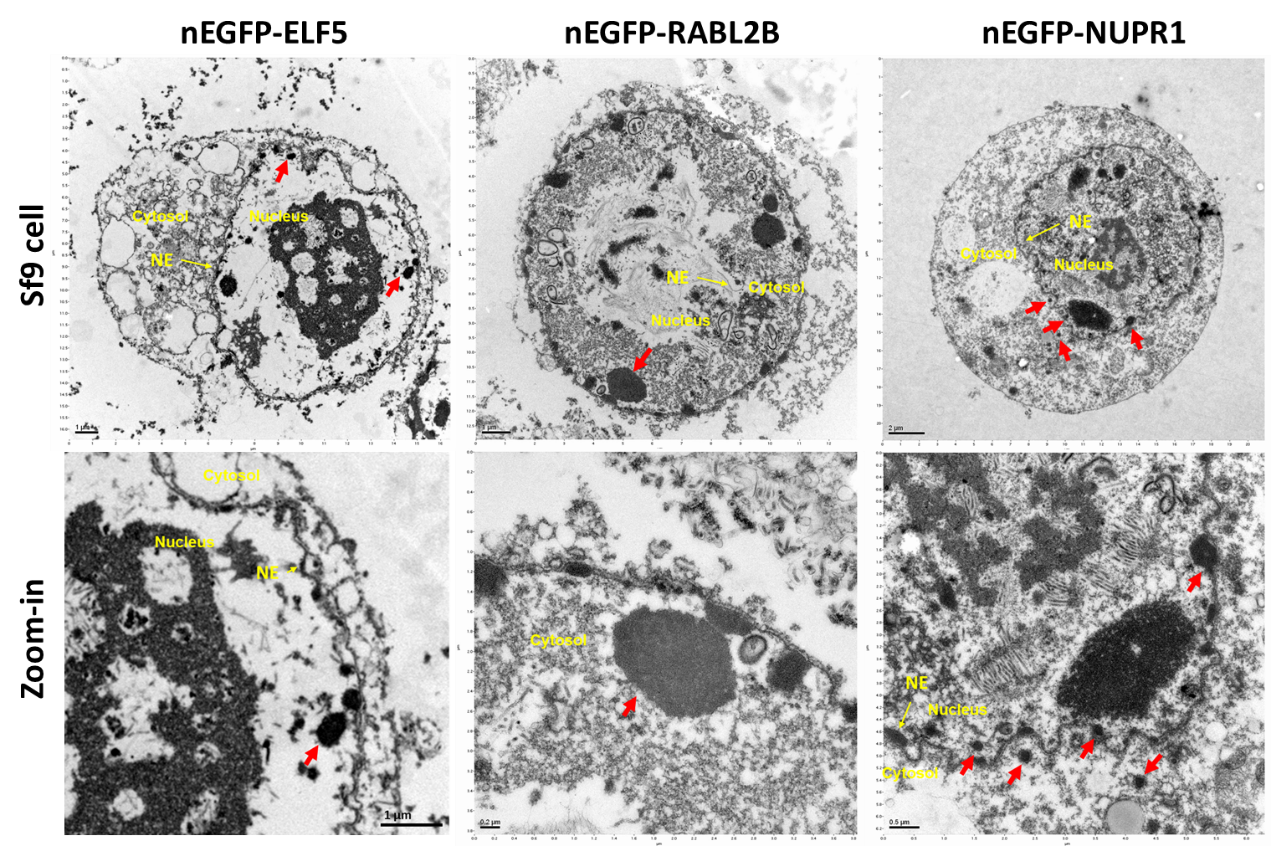


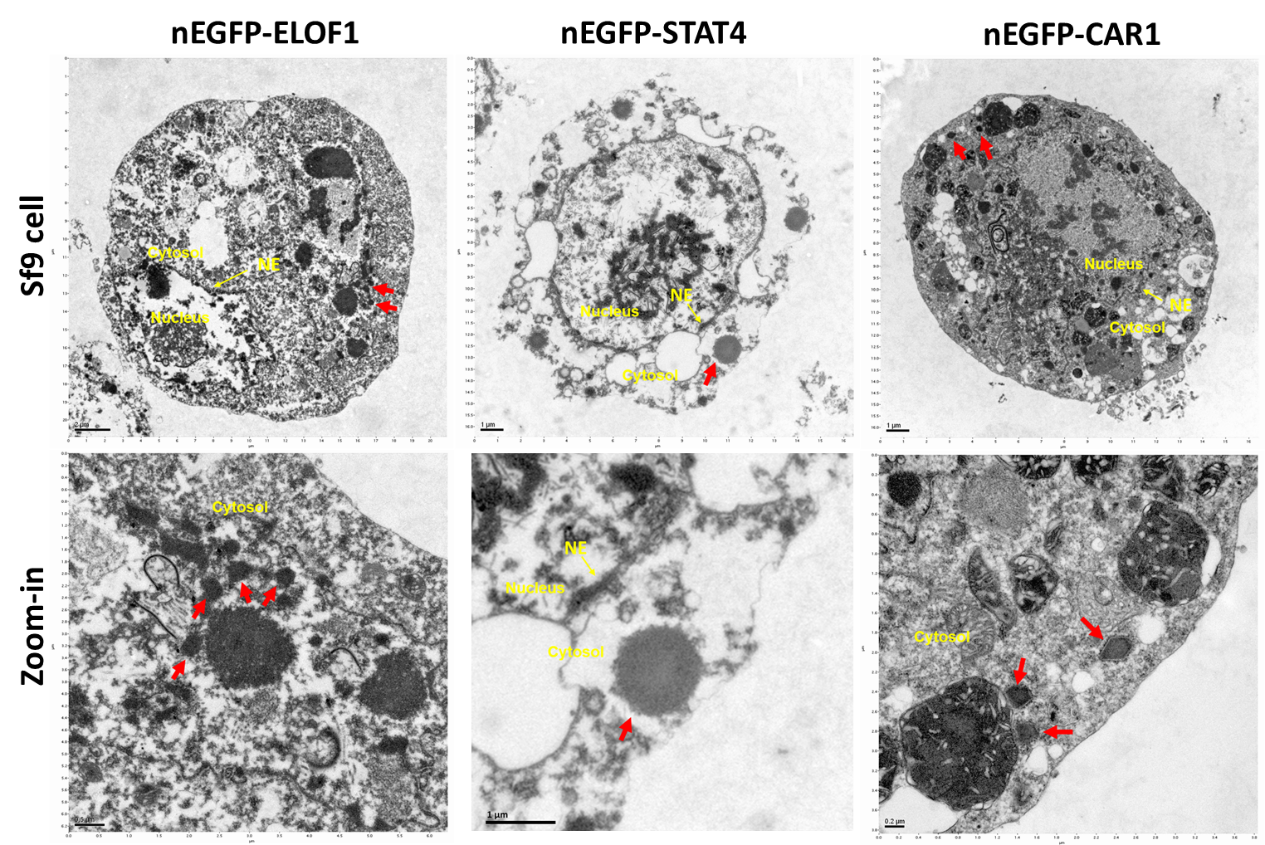


**Supplementary Figure S6. TEM of SONICC-positive hits.** Sf9 cells expressing the indicated SONICC-positive hits were examined by TEM to confirm the crystallinity of these targets. The lower panels include the zoom-in view of the Sf9 cells shown in the upper panel for each target. Non-sharp particles were observed for each target as indicated by the red arrows. Cell compartments (cytosol, nucleus, and nuclear envelope (NE)) have been highlighted for clarity in all images.

**
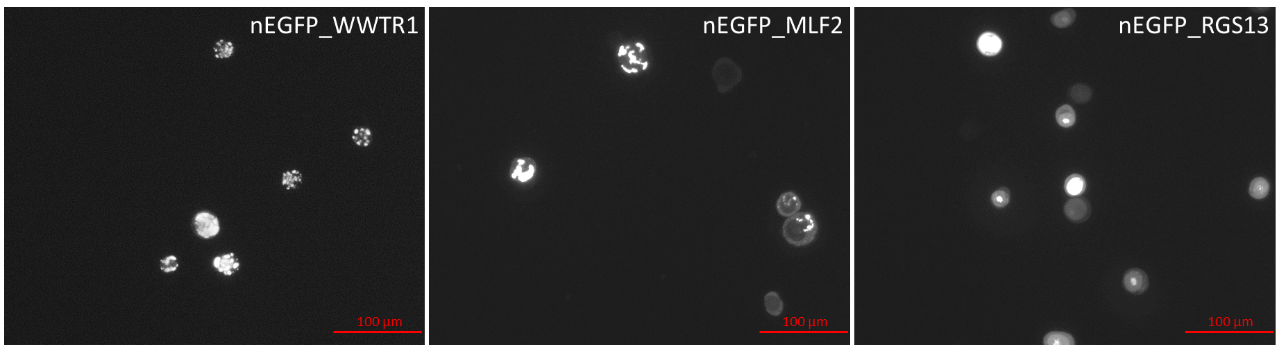
Supplementary Figure S7. UV fluorescence images of the indicated SONICC-positive hits.** The Sf9 cells expressing the indicated SONICC-positive hits showed different fluorescence patterns. Punctate fluorescence was observed for nEGFP-WWTR1 and nEGFP-MLF2 while uneven, diffuse fluorescence was observed for nEGFP-RGS13.
